# Structural and Functional Insights into the Mode of Action of a Universally Conserved Obg GTPase

**DOI:** 10.1371/journal.pbio.1001866

**Published:** 2014-05-20

**Authors:** Boya Feng, Chandra Sekhar Mandava, Qiang Guo, Jie Wang, Wei Cao, Ningning Li, Yixiao Zhang, Yanqing Zhang, Zhixin Wang, Jiawei Wu, Suparna Sanyal, Jianlin Lei, Ning Gao

**Affiliations:** 1 Ministry of Education Key Laboratory of Protein Sciences, Center for Structural Biology, School of Life Sciences, Tsinghua University, Beijing, China; 2 Department of Cell and Molecular Biology, Uppsala University, BMC, Uppsala, Sweden; 3 Tsinghua-Peking Joint Center for Life Sciences, Tsinghua University, Beijing, China; Brandeis University, United States of America

## Abstract

Obg proteins are a family of P-loop GTPases, conserved from bacteria to human. The Obg protein in *Escherichia coli* (ObgE) has been implicated in many diverse cellular functions, with proposed molecular roles in two global processes, ribosome assembly and stringent response. Here, using pre-steady state fast kinetics we demonstrate that ObgE is an anti-association factor, which prevents ribosomal subunit association and downstream steps in translation by binding to the 50S subunit. ObgE is a ribosome dependent GTPase; however, upon binding to guanosine tetraphosphate (ppGpp), the global regulator of stringent response, ObgE exhibits an enhanced interaction with the 50S subunit, resulting in increased equilibrium dissociation of the 70S ribosome into subunits. Furthermore, our cryo-electron microscopy (cryo-EM) structure of the 50S·ObgE·GMPPNP complex indicates that the evolutionarily conserved N-terminal domain (NTD) of ObgE is a tRNA structural mimic, with specific interactions with peptidyl-transferase center, displaying a marked resemblance to Class I release factors. These structural data might define ObgE as a specialized translation factor related to stress responses, and provide a framework towards future elucidation of functional interplay between ObgE and ribosome-associated (p)ppGpp regulators. Together with published data, our results suggest that ObgE might act as a checkpoint in final stages of the 50S subunit assembly under normal growth conditions. And more importantly, ObgE, as a (p)ppGpp effector, might also have a regulatory role in the production of the 50S subunit and its participation in translation under certain stressed conditions. Thus, our findings might have uncovered an under-recognized mechanism of translation control by environmental cues.

## Abstract 2

Kinetics and cryo-electronmicroscopy data provide insights into GTPase ObgEâ€™s role as a ribosome anti-association factor that is modulated by nutrient availability, coupling growth control to ribosome biosynthesis and protein translation.

## Author Summary

GTPases commonly act as molecular switches in biological systems. By oscillating between two conformational states, depending on the type of guanine nucleotide bound (GTP or GDP), GTPases are essential regulators of many aspects of cell biology. Additional levels of regulation can be acquired through the synthesis of other guanine nucleotide derivatives that target GTPases; for instance, when nutrients are limited, bacterial cells produce guanine tetraphosphate/pentaphosphate—(p)ppGpp—as part of the “stringent response” to adjust the balance between growth and survival. ObgE is a GTPase with many reported cellular functions that include ribosome biogenesis, but none of its functions is understood at the molecular level. Here we characterize, both biochemically and structurally, the binding of ObgE to its cellular partner, the 50S ribosomal subunit. Our results show that ObgE is an anti-association factor, which binds to the 50S subunit to block the formation of the 70S ribosome, thereby inhibiting the initiation of translation. Furthermore, the binding and anti-association activities of ObgE are regulated by guanine nucleotides, as well as by (p)ppGpp. We thus propose that ObgE is a checkpoint protein in the assembly of the 50S subunit, which senses the cellular energy stress via levels of (p)ppGpp and links ribosome assembly to other global growth control pathways.

## Introduction

Families of small P-loop GTPases are universally employed as molecular switches in all domains of life [1]. In *E. coli* there are ∼20 known GTPases, and most of them are involved in cellular processes related to ribosome functions. Among them, ObgE (Obg in *E. coli*, also known as CgtA or YhbZ) belongs to the highly conserved Obg GTPase family (*spo0B*-associated GTP-binding protein). Obg proteins are essential for cell growth in all tested bacterial species ([2] and references therein). The ObgE homologs are also widely present in eukaryotic organelles, including both chloroplasts and mitochondria. Despite extensive genetic studies of Obg proteins in several bacterial species, the exact molecular role of this protein family is still unclear. On the cellular level, it has been implicated in a variety of regulatory events in different species, including cell cycle control, DNA replication, stress response, sporulation, morphological development, and ribosome assembly (reviewed in [2],[3]). The pleiotropic phenotypes of Obg proteins indicate that this protein family might participate in certain essential processes that are central to various cellular functions.

In line with this view, ObgE and its homolog in *Vibrio cholera* have been implicated in the (p)ppGpp-mediated stringent response [4]–[6]. The ppGpp (guanosine tetraphosphate) and pppGpp (guanosine pentaphosphate), collectively called as (p)ppGpp, are important secondary messengers in bacteria and plants, regulating many cellular activities in response to various environmental stresses (reviewed in [7],[8]) by targeting transcription factors, GTPases, as well as proteins in nucleotide and lipid metabolism [9]. The first evidence implicating Obg in the (p)ppGpp pathway was from the crystal structure of the *Bacillus subtilis* Obg protein, in which a ppGpp molecule was found in the active site of its GTPase domain (GD) [10]. Later, it was shown that ObgE binds to ppGpp with a physiological affinity *in vitro* [4], and perturbation of ObgE function by different non-lethal mutations affects the total level of (p)ppGpp or the relative ratio of pppGpp/ppGpp during different growth phases [4],[5]. Furthermore, ObgE and its *V. cholera* homolog were found to co-purify and interact with SpoT [5],[6],[11], the cellular enzyme responsible for hydrolyzing (p)ppGpp to GTP or GDP [12], postulating a possible role of ObgE on (p)ppGpp degradation by SpoT. These observations have established a link between Obg proteins and the regulation of (p)ppGpp, and suggested that many phenotypes associated with mutant Obg proteins might originate from impaired temporal control of the cellular (p)ppGpp level.

In the meantime, converging biochemical evidences show that Obg proteins also physically interact with the ribosome or ribosomal subunits; the observations include bacteria *Vibrio harveyi*, *E. coli*, *Caulobacter crescentus*, *Salmonella typhimurium*, *B. ubtilis*, *Chlamydia abortus*, and *Mycobacterium tuberculosis* (reviewed in [3]). It was also revealed that mutations in ObgE lead to cellular defects in the 50S subunit maturation [13],[14]. Moreover, Obg proteins from *E. coli* and *S. typhimurium* were shown to have physical [15] or genetic interaction [16] with 23S rRNA modification enzymes. On the basis of these data, a primary role of ObgE in the 50S subunit maturation was proposed [13],[14]. Given the absolute requirement of ribosome function for all cellular activities, this provides an alternative explanation of pleiotropic phenotypes of Obg proteins in multiple disparate cellular events.

In the present work, we demonstrate that ppGpp enhances the binding of ObgE to the 50S subunit and promotes dissociation of the 70S ribosome into subunits. Interestingly, we find that ObgE plays an important role as a 50S based anti-association factor, which inhibits the formation of 70S ribosomes from the naked subunits as well as from an mRNA programmed 30S-preinitiation complex (30S-preIC). The inhibition in subunit association also leads to a slower dipeptide formation when ObgE is bound to the 50S subunit. Importantly, a C-terminus deleted construct of ObgE (ObgE-NG), containing the N-terminal domain (NTD) and the central GD, is sufficient for its anti-association activity, suggesting that the NTDs constitute the main activity center of ObgE. Next, we solved the cryo-EM structure of the 50S subunit bound with ObgE. Structural data reveal that the NTD of ObgE is a structural mimic of the A-site tRNA, which exhibits specific interactions with the ribosomal peptidyl-transferase center. Together with previously published data, our results suggest that ObgE might be an important player in the (p)ppGpp regulatory circuit, regulating the assembly of the 50S subunit, and blocking the subunit association and further downstream events in protein translation in response to signal of the nutrient availability.

## Results

### ppGpp Stimulates the Binding of ObgE to the 50S Subunit

**Figure 1:**
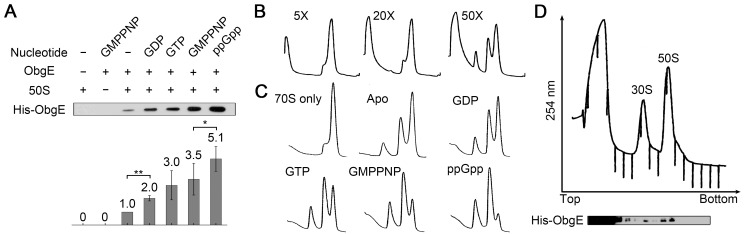
ppGpp stimulates the 50S subunit binding and 70S ribosome dissociation activities of ObgE. (A) Co-sedimentation assay on the binding of ObgE (1 µM) to the 50S subunit (1 µM), in the absence or presence of GDP, GTP, GMPPNP, or ppGpp (0.5 mM). Pellets were resolved by SDS-PAGE and examined by Western blot analysis (anti-His antibody). Quantification was performed by adjusting the value of the apo state to 1.0. Error bars (standard deviation) were calculated from three independent experiments. (B) Dissociation of 70S ribosomes (1 µM) by varying amount of ObgE (from 5- to 50-fold excess), in the presence of GDP (2 mM). (C) Dissociation of 70S ribosomes (1 µM) by 30-fold excess of ObgE, in the absence or presence of GDP, GTP, GMPPNP, or ppGpp (2 mM). (D) Complete dissociation of 70S ribosomes (1 µM) by 50-fold excess of ObgE in the presence of GMPPNP (2 mM). Fractions were resolved by SDS-PAGE and examined by Western blot analysis.

Previous studies showed that endogenous ObgE co-fractionates mainly with the 50S fraction [5],[11],[13],[14], In addition, GTP or GMPPNP was proven to stimulate the binding of ObgE to the 50S subunit [13] or to the 23S rRNA [14]. However, it is not clear whether (p)ppGpp could also modulate the binding of ObgE to the 50S subunit. To test this possibility, we performed co-sedimentation assay to examine the binding of ObgE to the 50S subunit in the presence of different nucleotides. Although ObgE in the apo state showed weak binding to the 50S subunit, addition of guanine nucleotides significantly enhanced its binding to the 50S subunit (Figure 1A). Especially, ppGpp increased the occupancy of ObgE on the 50S subunit by over 5-fold, compared to the apo state. In addition, similar to other ribosome-interacting GTPases, ObgE showed a higher affinity to the 50S subunit in the presence of GTP or GMPPNP than GDP (Figure 1A).

These observations indicate that ppGpp binding, similar to GDP and GTP, indeed influences the interaction between ObgE and the 50S subunit. The marked effect of ppGpp, as well as the subtle difference with other nucleotides, on the 50S binding ability of ObgE, suggests that ObgE might sense changes in the nucleotide pool during different growth phases and adjust its behavior accordingly.

### ObgE Promotes the Dissociation of 70S Ribosomes

Previously, it was shown that overexpression of the plasmid-encoded ObgE leads to the co-fractionation of the protein with the 70S, as well as polysome fractions [5]. We then sought to examine whether ObgE could bind to the 70S ribosome *in vitro*. However, unexpectedly, ObgE was found incompatible with the 70S ribosome when added in excess, and the latter was disassembled into subunits (Figure 1B and 1C). Upon subunit dissociation, ObgE remained associated with the separated 50S subunits (Figure 1D). Importantly, this dissociation of 70S ribosomes did not seem to require energy input from GTP-hydrolysis, because both GDP and ppGpp also enabled this 70S-spliting activity (Figure 1C). As illustrated in Figure 1B, the 70S dissociation was dependent on ObgE concentration. Further comparison of the extent of 70S disassembly in the presence of different nucleotides indicates that the 70S dissociation activity of ObgE is well correlated with its 50S binding ability (Figure 1A). As expected from the results presented in Figure 1A, the maximal dissociation was observed when ObgE was saturated with ppGpp (Figure 1C).

Obg proteins from different species contain very diverse C-terminal domains (CTDs), connected to the GDs by long flexible linkers [3],[17]. We therefore tested whether the CTD plays any role for the dissociation of 70S ribosomes. As shown in Figure S1, deletion of the CTD does not impair the binding of ObgE-NG to the 50S subunit (Figure S1A), and ObgE-NG is sufficient to promote dissociation of 70S ribosomes in a way similar to full-length ObgE; the maximal dissociation is seen in the presence of ppGpp (Figure S1B and S1C).

Altogether, these data indicate that ObgE binds to the 50S subunit preferentially over the 70S ribosome, and the association is likely mediated by its conserved NTD. Most likely, by binding to the 50S subunit ObgE prevents the re-association of the 30S subunit thereby shifting the 70S dissociation equilibrium toward free subunits in the steady-state.

### ObgE Is a 50S Based Anti-association Factor

**Figure 2:**
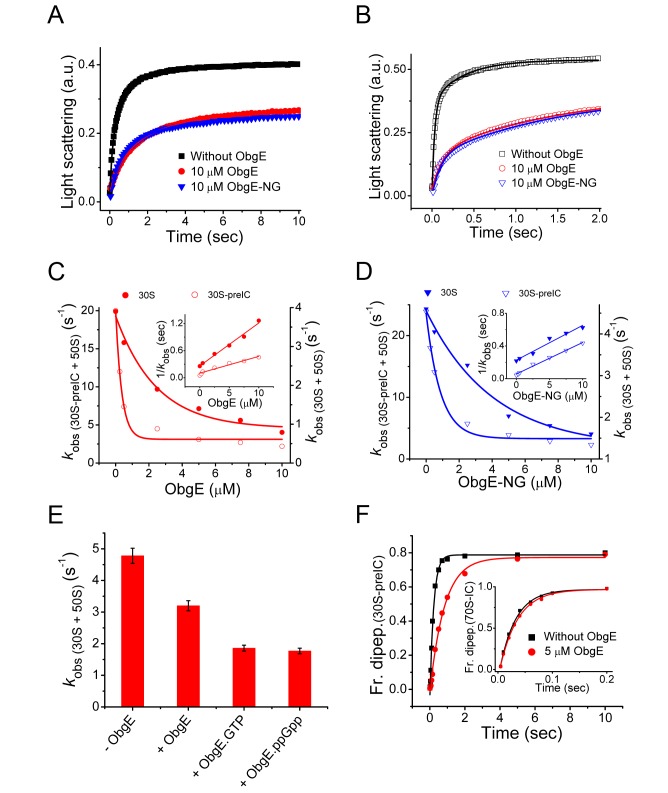
ObgE is a 50S based anti-association factor. (A) Formation of 70S ribosome by association of naked 30S subunit (0.25 µM) with 50S subunit alone (0.25 µM) (black square), or the 50S subunit preincubated with ObgE (10 µM) (red circle) or ObgE-NG (10 µM) (blue triangle), followed by Rayleigh light scattering with time in a stopped flow apparatus. The solid lines are drawn by fitting the data according to the subunit association model described in [59]. (B) Same as (A), except that the 30S-preIC (0.25 µM) was used. (C) The observed rates of subunit association (*k*_obs_) with naked 30S (filled circle, scale on the right vertical axis) and the 30S-preIC (open circle, scale on the left vertical axis), with increasing concentration of ObgE. Inset, the reciprocal of *k*_obs_ plotted as a function of ObgE concentration, fitted with the straight line equation. (D) Same as (C), except that ObgE-NG was used. (E) The rates of association of the naked subunits in the presence of ObgE with various guanine nucleotides. (F) The formation of f[^3^H]Met–Leu dipeptide without (black square) or with (red circle) ObgE (5 µM) starting from the 30S-preIC. The solid lines are drawn by fitting the data with single exponential function. The inset shows the f[^3^H]Met–Leu dipeptide formed from the 70S-IC without (black square) or with (red circle) ObgE (5 µM).

We have studied the effect of ObgE in ribosomal subunit association using naked 30S subunits or an mRNA programmed 30S-preinitiation complex (30S-preIC) containing fMet-tRNA^fMet^ in the P-site. The kinetics of subunit association was followed by monitoring the increase in light scattering after rapid mixing of the 30S (or 30S-preIC) and 50S subunits in a stopped-flow instrument. In both cases, the rates of subunit association, in the absence of ObgE, matched closely with previously published results [18],[19]. ObgE, as expected from its preferential binding to the 50S subunit, showed a strong inhibition on the subunit association both for naked 30S (Figure 2A) and 30S-preIC (Figure 2B). The rates of the subunit association [(*k*_obs 30S_  = 4±1 s^−1^) and (*k*_obs 30S-preIC_  = 20±0.2 s^−1^)] decreased gradually with increasing concentration of ObgE (Figure 2C). Subsequently, the mean time for subunit association, estimated as the reciprocal of the observed rate (1/*k*_obs_), increased linearly with ObgE concentration (inset in Figure 2C), thereby suggesting that ObgE competitively inhibits the 30S subunit for binding to the 50S subunit. By comparing the concentration of ObgE required for half-maximal inhibition (Figure 2C), it is evident that the ObgE-mediated inhibition is stronger in the 30S-preIC association than the naked subunit association. For the 30S-preIC association the *Ki* is around 0.3 µM, while the same for naked 30S association is 2 µΜ. Highly consistent with full-length ObgE, the C-terminal deleted ObgE-NG also showed inhibition of subunit association in an extent comparable to ObgE (Figure 2A and 2B). For ObgE-NG the estimated *Ki* values for inhibition of 30S-preIC and naked 30S association are 1 and 3.7 µΜ, respectively (Figure 2D). The inhibition was more profound in the presence of guanine nucleotides with ObgE, both GTP and ppGpp led to higher inhibition of subunit association (Figure 2E), as expected from the higher affinity of ObgE to the 50S subunit in the presence of these guanine nucleotides (Figure 1A). Thus, our results demonstrate that ObgE blocks subunit association and hence translation initiation by binding to the 50S subunit, and the NTD of ObgE is likely the main activity center.

We have further tested the effect of ObgE in dipeptide synthesis starting from 30S-preIC or 70S-initiation complex (70S-IC). The formation of fMet-Leu (ML) dipeptide starting from 30S-preIC required about 200 msec (*k*_obs dipep 30S-preIC_  = 4.7±0.2 s^−1^), which involved subunit association followed by peptide bond formation. In the presence of ObgE with 50S containing elongation mix (see Materials and Methods for details), the rate of dipeptide formation slowed down about four times (Figure 2F, *k*_obs dipep 30S-preIC obgE_  = 1.2±0.05 s^−1^). However, ObgE did not show any effect on the rate of dipeptide formation when 70S-IC was previously associated and ObgE was added with the ternary complex (*k*_obs dipep 70S-IC_  = 33±2 s^−1^ and *k*_obs dipep 70S-IC ObgE_  = 30.3±1 s^−1^) (Figure 2F, inset). Thus, our results suggest that the ObgE has no effect on peptide bond formation. The defect seen in dipeptide formation starting from 30S-preIC was due to its anti-association activity. When ObgE is bound to the 50S subunit it blocks association and consequently the downstream steps in protein synthesis get inhibited.

Following these observations, we tested the effect of ObgE on translation in a multiple turn-over reaction. As expected, ObgE inhibits the *in vitro* translation of a reporter gene in a dose-dependent manner (Figure S2). Consistent with the *in vitro* data, overexpression of ObgE in *E. coli* cells leads to a slower growth (Figure S3A and S3B) and a substantial increase of free 50S fractions in the ribosome profile (Figure S3C and S3D).

Thus, both our *in vitro* and *in vivo* results suggest that ObgE acts as an anti-association factor. The role of IF3 as an anti-association factor is well-known [20], which binds primarily to the 30S subunit and prevents premature association of the ribosomal subunits. We show that ObgE demonstrates a similar anti-association activity by binding to the 50S subunit.

### Cryo-EM Structure of the 50S Subunit Bound with ObgE·GMPPNP

**Figure 3:**
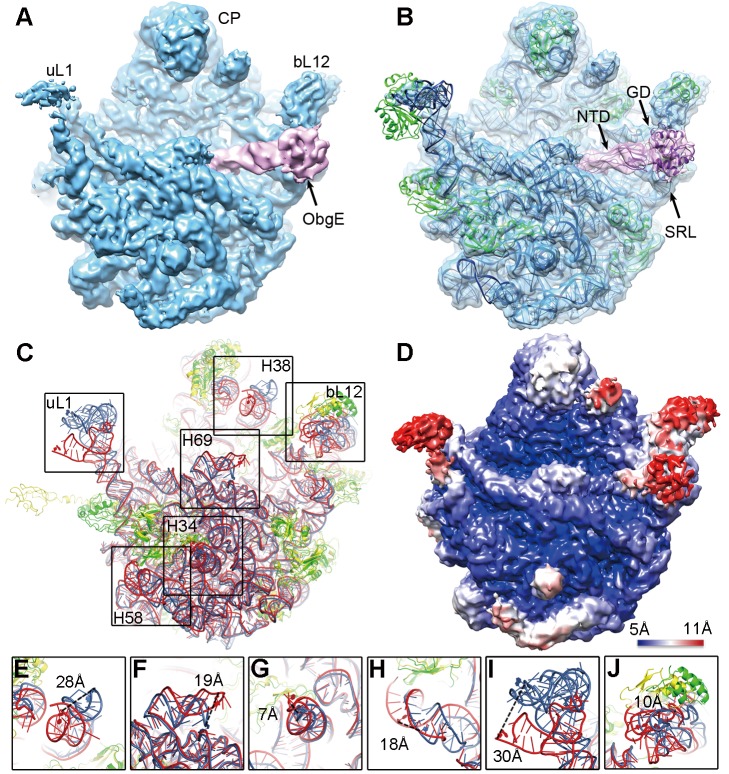
Cryo-EM structure of the 50S·ObgE·GMPPNP complex. (A) The cryo-EM map is displayed in surface representation, with the 50S subunit and ObgE colored blue and pink, respectively. (B) The atom model of the 50S·ObgE·GMPPNP complex is displayed in cartoon representation, and superimposed with the density map. Ribosomal RNA, ribosomal proteins, and ObgE are colored blue, green, and purple, respectively. CP, central protuberance; uL1, uL1 stalk; bL12, bL12 stalk. (C) Crystal structure of the 50S subunit [71] (rRNA in red and r-proteins in yellow) is superimposed with the atomic model of the 50S·ObgE·GMPPNP complex (rRNA in blue and r-proteins in green). (D) Local resolution map of the 50S·ObgE·GMPPNP structure. (E–J) Close-up views of regions with large scale conformational changes as boxed in (C). Distances between selected residue pairs are represented in dashed lines.

We next determined the cryo-EM structure of the 50S subunit bound with ObgE·GMPPNP. The cryo-EM density map was solved at a nominal resolution of 5.5 Å. As shown in Figure 3, ObgE binds to the intersubunit face of the 50S subunit, at a position commonly used for the docking of translational GTPases. Specifically, the NTD of ObgE protrudes into the peptidyl-transfer center (PTC), and its GD is situated between the bL12 (adopted after a newly proposed ribosomal protein naming system described in [21]) stalk base and the sarcin-ricin loop (SRL) of the 23S rRNA (Figure 3A and 3B). However, we did not find extra densities that could be attributed to the CTD of ObgE, indicating that this domain is highly flexible. Structural superimpositions of ObgE with four translational GTPases (IF2, EF-Tu, EF-G, and RF3) on the 50S subunit all report a large steric clash (Figure S4), indicating that the binding of ObgE is incompatible with these translation factors on the 50S or 70S ribosomes.

Both the 50S subunit and ObgE undergo conformational changes upon the complex formation. Compared with crystal structures of Obg proteins [10],[17], a large scale rotation between the NTD and GD (Figure S5) is necessary to assume the 50S bound conformation. On the 50S subunit side, significant conformational changes are seen on uL1 stalk, bL12 stalk, helix 38, helix 34, helix 58, as well as helix 69 of the 23S rRNA (Figure 3C), all localized in the intersubunit face. These changes are well correlated with the local resolution map of the 50S·ObgE complex (Figure 3D). Very interestingly, upon binding to ObgE, helix 69 is seen to have a massive movement by about 19 Å (Figure 3C and 3F), which, if mapped on the 70S ribosome structure, would directly disrupt a strong intersubunit bridge (B2a). The B2a is essential for intersubunit association to form the 70S ribosome [22], and ribosome recycling factor (RRF) employs exactly the same mechanism to break the B2a during the ribosome recycling [23]. This large movement of helix 69, as well as the observed conformational changes on bridges B1a (helix 38) (Figure 3C and 3E) and B4 (helix 34) (Figure 3C and 3G), perfectly explains the anti-association activity of ObgE.

Another intriguing conformational change takes place on the NTD of uL11. Upon the binding of ObgE, the uL11-NTD becomes “invisible” in the cryo-EM density map (Figure S6). The flexibility of the uL11-NTD probably results from the interaction between the ObgE-GD and the bL12 stalk base (H43-44).

### Specific Polar Interactions between the ObgE-NTD and the PTC

The ObgE-NTD is composed of an eight-stranded β-barrel base and a unique glycine-rich protrusion containing six left-handed helices of the poly-Pro type II conformation (Figure S5) [10],[17]. At the tip of the protrusion, connecting the six helices are three loops (Figure S5B). These loops are conserved in both sequence and length among Obg family proteins (Figure S7).

**Figure 4:**
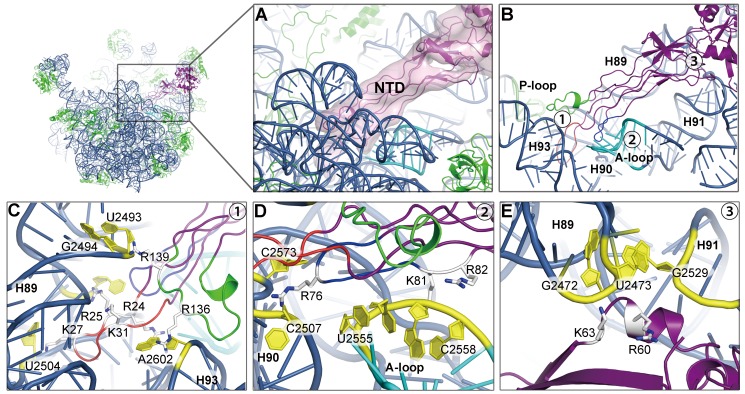
Interaction of the ObgE-NTD with the peptidyl transferase center. (A) Binding position of the ObgE-NTD on the 50S subunit, with the ObgE density map displayed in transparent surface representation. (B) Overview of the interactions of loop 1 (red), loop 2 (dark blue), and loop 3 (green) of the ObgE-NTD with H89, H90, H91, H93, A-loop (cyan), and P-loop (lime) of the 23S rRNA. (C–E) Close-up views of numbered locations (1–3) in (B), with conserved basic residues of the ObgE-NTD highlighted in stick model. For illustration, orientations of (C–E) are set differently from that of (B). (C) Interactions of Loop 1 and Loop 3 with H89 and H93. (D) Interaction of Loop 2 with H90 and the A-loop. (E) Interaction of the ObgE-NTD β-barrel base with H89 and H91. Residues of ObgE are shown in stick representation and colored white, and residues of the 23S rRNA are shown in cartoon representation and colored yellow.

**Figure 5:**
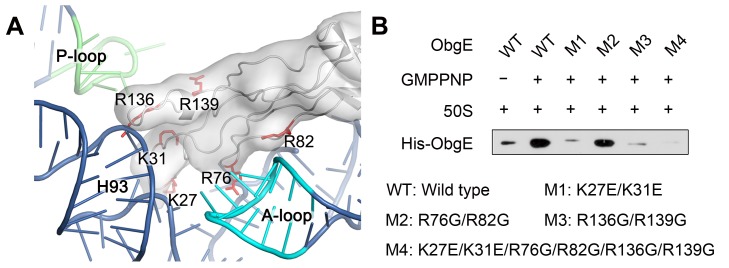
Conserved basic residues in the ObgE-NTD are required for the 50S binding. (A) Positions of conserved arginine or lysine residues that are subjected to site-directed mutagenesis. (B) Co-sedimentation assay on the binding of various ObgE mutants to the 50S subunit. Wild type (WT) and mutant ObgE (M1–M4) were incubated with equal amount of 50S subunits in the absence or presence of saturating GMPPNP and subjected to co-sedimentation assay. M1, M2, M3, and M4 refer to ObgE mutants, K27EK31E, R76GR82G, R136GR139G, and K27EK31E R76GR82G R136GR139G, respectively.

The binding environment of the ObgE-NTD on the 50S subunit is exclusively in rRNA helices (Figure 4), including helix 89, helix 90, helix 91, helix 93, and the A-loop (helix 92). Consistent with our structural model, most of the conserved lysine and arginine residues in the ObgE-NTD are located at the rRNA interface (Figures 4C–4E and S7). Specifically, the tip of the NTD shows tight polar interaction with the PTC (Figure 4C and 4D). At the opposite end, a short helical insertion from the β-barrel base is also seen to interact with the junction between helix 89 and helix 91 (Figure 4E). To be more specific, several highly conserved basic residues from the three intervening loops, including R24, R25, K27, and K31 from loop 1; R76, K81, and R82 from loop 2; and R136 and R139 from loop 3, are within 4 Å distance from a number of the PTC residues, such as U2493, G2494, U2504, A2602, C2573, U2555, C2558, and C2507 (Figure 4). To confirm these structural observations, we introduced mutations to a few selected arginine or lysine residues on the three loops and tested the binding of ObgE mutants to the 50S subunit (Figure 5). As a result, all of the mutations impaired the binding, and especially, loop 1 (K27EK31E) and loop 3 (R136GR139G) mutants exhibited almost abolished binding activity.

The sequence (Figure S7) and mutational data (Figure 5) indicate that the polar interactions between the ObgE-NTD and the PTC are highly likely very specific and conserved across species, which suggests that the recognition of the PTC is a universal function for the NTDs of all Obg proteins.

### The ObgE-NTD Is a Structural Mimic of the Acceptor Arm of the A-Site tRNA

**Figure 6:**
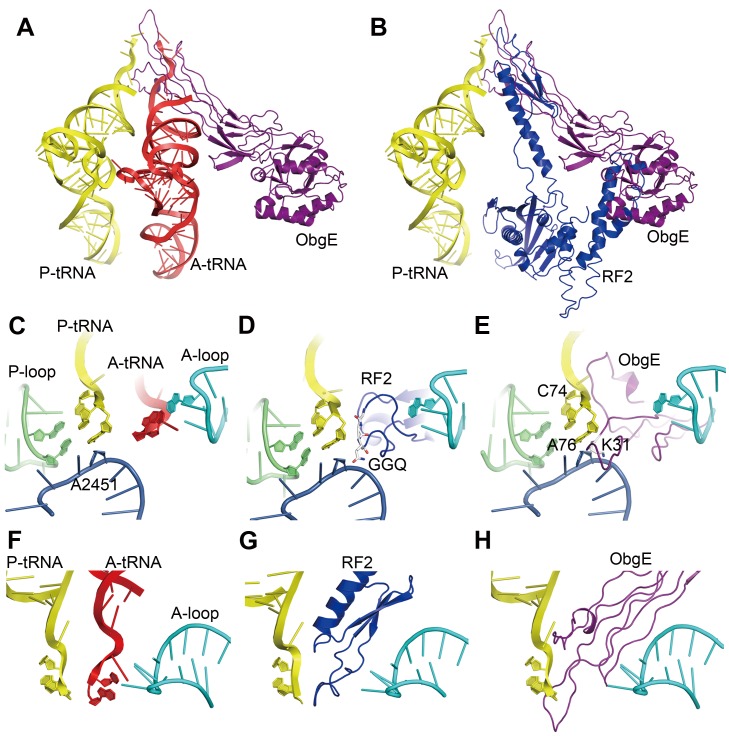
Comparison of ObgE with the A-site tRNA and release factor 2 on the 50S subunit. (A) Superimposition of ObgE (purple) with the A-site tRNA (red) and the P-site tRNA (yellow) [PDB 2WDK and 2WDL, [75]]. (B) Superimposition of ObgE with the P-site tRNA and RF2 (dark blue) from the crystal structure of a release complex [PDB 2X9S and 2X9R, [28]]. (C–E) Close-up views of the CCA-end of the P-site tRNA, with the A-site tRNA (C), RF2 (D), and ObgE (E) superimposed. (F–H) Close-up views of the CCA-end of the A-site tRNA (F), GGQ-motif of RF2 (G), and the NTD protrusion of ObgE (H), showing local specific interactions with the ribosomal A-loop. The A-loop, P-loop, and H92 (A2451) of the 23S rRNA are colored cyan, lime, and blue, respectively. Critical residues of RF2 and ObgE are shown in stick representation and labeled accordingly.

Interestingly, the loop regions of the ObgE-NTD occupy the space that accommodates the acceptor arm of the A-site tRNA (Figure 6A). A comparison with the crystal structure of the 70S·RF2 complex [24] indicates that the tip of the ObgE-NTD overlaps exactly with the GGQ-motif containing domain of RF2 (Figure 6B). A close inspection at the PTC suggests that the two factors employ a very similar way to interact with this functional center (Figure 6C–6H). Specifically, when the P-site tRNA is superimposed with the 50S·ObgE structure, residues I29–K31 from loop 1 of ObgE are capable of forming interaction with CCA-end of the P-site tRNA, and K31 is inserted between A76 of the P-site tRNA and A2451 of the 23S rRNA (Figure 6E), displaying an astonishing resemblance to the GGQ-motif of RF2 (Figure 6D). Besides the possible interaction with the P-site tRNA, the ObgE-NTD also interacts with the A-loop of the 23S rRNA, in a strikingly similar way as RF2 does (Figure 6G and 6H).

The structural similarity between the acceptor arm of a tRNA and the NTD of ObgE indicates that like many translation factors, ObgE also adopts a tRNA mimicry strategy to interact with the ribosome.

### ObgE Is a Distinctive Member of the Ribosome-dependent GTPases

**Figure 7:**
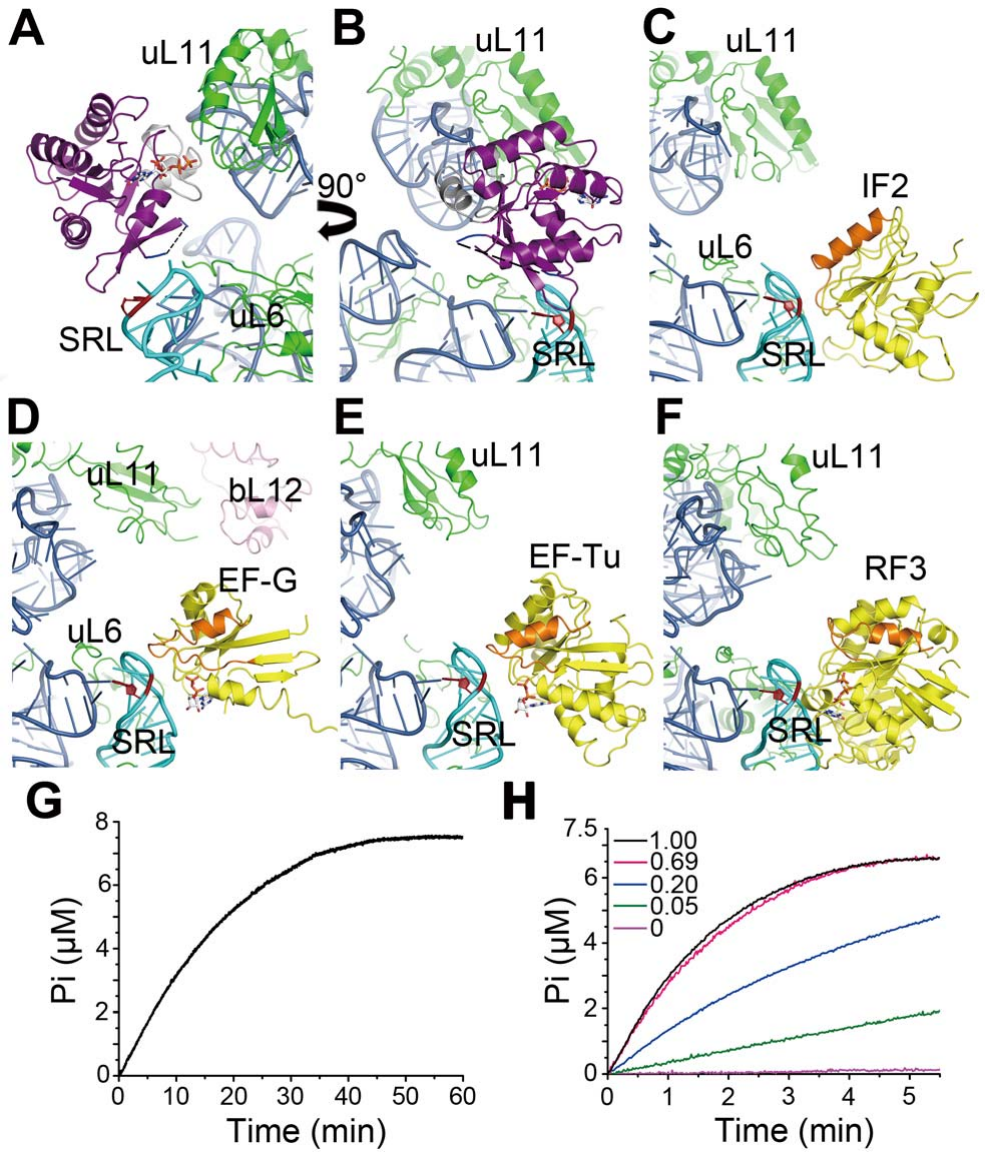
ObgE is a distinctive 50S-dependent GTPase. (A and B) Two different views to illustrate the interaction of the ObgE-GD (purple) with the 50S subunit. Interactions of the GDs of IF2 (C) [PDB 1ZO3, [25]], EF-G (D) [PDB 2WRI and 2WRJ, [26]], EF-Tu (E) [PDB 2WRN and 2WRO, [27]], and RF3 (F) [PDB 3SFS and 3SGF, [29]] with the 50S subunit, shown in the same view as (B). The 23S rRNA, r-proteins, ObgE, and translational GTPases are colored blue, green, purple, and yellow, respectively. Switch I of ObgE is disordered and is shown by dashed lines. Switch II of ObgE, Switch II of other factors, SRL, and A2662 are colored, grey, orange, cyan, and red, respectively. Guanine nucleotides are shown in stick models. (G) Low intrinsic GTPase activity of ObgE, monitored by inorganic phosphate production. (H) Time-course GTP hydrolysis by ObgE, in the presence of indicated amount of purified 50S subunits (0.05-, 0.2-, 0.69-, and 1.0-fold).

The GDs of classical translational GTPases, such as IF2 [25], EF-G [26], EF-Tu [27], and RF3 [28],[29], all show only limited contact with the bL12 stalk base (containing uL11, H43, and H44) on the ribosome (Figure 7). Unlike these, the ObgE-GD itself interacts directly with the bL12 stalk base, and bridges the gap between SRL and bL12 stalk base (Figures 7A and S6). Other than that, compared with translational GTPases, the ObgE-GD is distinctively orientated on the 50S subunit, which places the Switch regions and the nucleotide binding pocket of the GD rather distant from the conserved A2662 of the SRL (Figure 7A and 7B).

This unprecedented placement of the ObgE-GD on the 50S subunit immediately raises the question whether the 50S subunit serves as the GTPase-activating protein (GAP) for ObgE, as does for classical translational GTPases. Therefore, we performed a GTPase activity assay on ObgE in the presence or absence of purified 50S subunits. As expected, the basal GTPase level of ObgE is relatively low, similar to previous measurements [4],[11],[16]. The hydrolysis rate in the absence of the 50S subunit is ∼0.0268 min^−1^; with a sufficient time ObgE is capable of converting all GTP to GDP (Figure 7G). In the presence of increasing amounts of 50S subunits, the phosphate production is accelerated accordingly (Figure 7H). When supplied with equal amount of 50S subunits, the hydrolysis rate is stimulated by about 120-fold, being about 3.15 min^−1^.

This moderate stimulation by the 50S subunit is in sharp contrast to translational GTPases, e.g., the GTP hydrolysis on EF-Tu and EF-G is enhanced by the 70S ribosome by over seven orders of magnitude [30]. More importantly, the binding and subsequent GTP hydrolysis of translational GTPases are regulated by the dynamic bL12 stalk on the ribosome in very distinct ways [19],[31]–[33]. It remains to be tested whether the bL12 stalk has any role in activating the GTPase of ObgE. Nevertheless, it is clear that ObgE represents a novel class of ribosome-interacting GTPases, whose GTPase activation mechanism should be different from those of classical translational GTPases.

## Discussion

### Molecular Role of ObgE in the 50S Subunit Assembly

In the present work, we reveal that the interaction between the PTC and the evolutionarily conserved NTD of ObgE is highly specific (Figures 4 and 5). This suggests that a primary molecular role of ObgE is directly related to the ribosome, which is consistent with the proposed role of ObgE in the ribosome assembly [13],[14].

The assembly function of ObgE started with a genetic study showing that overexpression of ObgE could suppress the slow growth and ribosome profile defect of the Δ*rrmJ* strain [16]. RrmJ is a 23S rRNA methyl-transferase (U2552 of the A-loop) required for late-stage 50S subunit assembly [34]. Later it was also shown that Obg homolog from *S. typhimurium* physically interacts with another 23S rRNA modification enzyme RluD (pseudouridine synthatase for ψ1911, ψ1915, and ψ1917 of helix 69) *in vitro* [15]. Consistently, pull-down experiment indicates ObgE also co-localizes with factors required for the 50S assembly, such as CsdA and DnaK [14]. Further analysis of the pre-50S subunits accumulated in an ObgE mutant (G80ED85N) strain [13] revealed several 50S maturation defects, including reduced binding of several 50S proteins (e.g., bL33, bL34, and uL16), impaired 23S rRNA processing, and prolonged association of RluC (pseudouridine synthase for ψ955, ψ2504, and ψ2580) and RrmJ with the pre-50S subunits.

**Figure 8:**
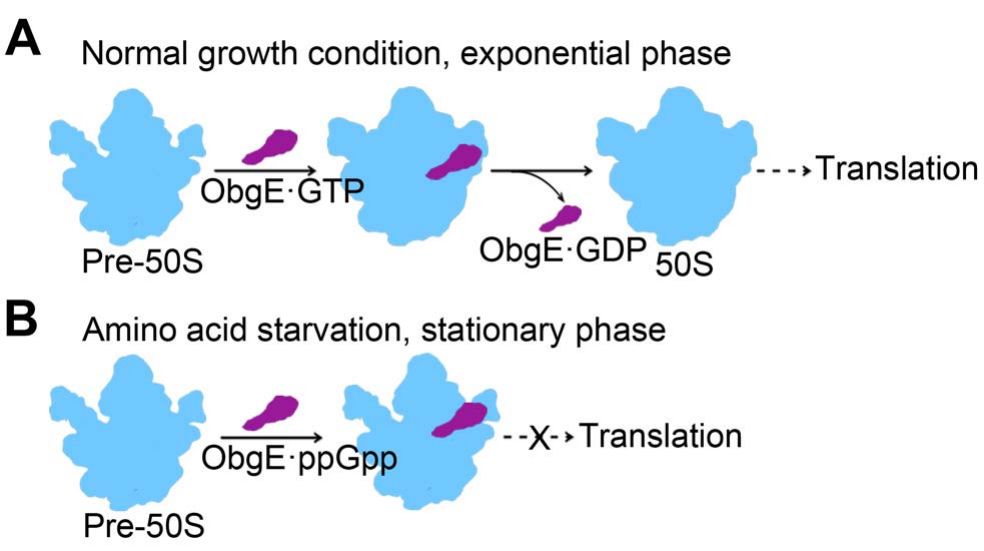
Proposed model for the function of ObgE in different growth phases. (A) In exponential phase of growth, ObgE functions primarily as a surveillance factor for the late-stage assembly the 50S subunit. In addition, it prevents the pre-50S subunit from premature association with the 30S subunit. (B) In stationary phase, ObgE acts as a ppGpp effector to delay the 50S maturation and sequesters large numbers of 50S subunits from being engaged in subunit association and thus downregulates global translation.

From our structural data, the binding position of ObgE is exactly next to the modification sites of RrmJ, RluD, and RluC (Figure S8). The release of ObgE from the 50S subunit, therefore, might mark the finish point of the 50S assembly, considering that ObgE might act very late in the assembly pathway [13]. In this sense, ObgE could be a checkpoint protein during the late-stage assembly, which monitors the modification status of these critical residues and local conformation of the PTC. The correctly modified and assembled 50S subunit then signals the GTP-hydrolysis and subsequent release of ObgE (Figure 8A). Escape from this quality control mechanism results in hypo-modified 50S subunits into the translation pool, leading to a profound impact on translation. For examples, lack of methylation at U2552 increases translation accuracy at the expense of efficiency [35], and deletion of *rluD* gene in *E. coli* results in a defect in translation termination, with an increased rate of stop codon read-throughs [36],[37]. Therefore, the reported diverse phenotypes, associated with various ObgE mutants, might be at least partially originated from subtle changes on cellular translatome profile, related to specific proteins in different cellular events.

The stronger interaction of ObgE with the 50S subunit in the presence of ppGpp (Figure 1), therefore, highly likely reflects another level of regulation on the ribosome assembly by (p)ppGpp during stringent response, which is to delay the 50S assembly by a prolonged association of ObgE·ppGpp with the pre-50S subunits, in addition to the well-known direct role of the (p)ppGpp on rRNA transcription [7]. When GTP is plenty in the mid-log phase, ObgE functions primarily as a 50S assembly factor to facilitate the 50S subunit maturation. In contrast, when the cells are challenged by nutrient limitation or enter stationary phase, the intracellular level of ppGpp sharply rises, and ObgE is dominantly modulated by ppGpp. As an effector, ObgE·ppGpp over-stays on the 50S subunits, and consequently, downregulates the subunit production (Figure 8B).

In the process of eukaryotic ribosome assembly, many 60S and 40S maturation factors also possess anti-association activity, and some of them have functional implications in translation initiation (reviewed in [38],[39]). One such example is a 60S subunit assembly factor eIF6 (Tif6 in yeast), which binds to the subunit interface of the pre-60S particles beside the SRL [40],[41] and blocks the subunit association and downstream initiation events. Mammalian eIF6 has also been shown to have a profound role in translation control (reviewed in [42]). Interestingly, in yeast the release of Tif6 from the pre-60S particles by an assembly GTPase Efl1 is triggered by the maturation state of the P-site on the 60S subunit [43]. Taken together, it is apparent that there are common quality control mechanisms in the assembly of bacterial and eukaryotic ribosomes, which make use of the maturation states of functional centers (such as PTC) on the subunits as structural checkpoints.

### The Anti-association Function of ObgE Plays Important Role in Stringent Response

Our biochemical results demonstrate that ObgE possesses anti-association activity in addition to its role in the 50S subunit maturation. By binding to the 50S subunit ObgE prevents association of the naked 30S subunit as well as the properly programmed 30S-preIC to the 50S subunit. Our structural analysis shows that ObgE obstructs the binding of the 30S subunit by inducing significant conformational changes at several intersubunit bridging contacts on the 50S subunit, including B1a, B2a, and B4 (Figure 3).

The anti-association activity of ObgE can have deep implications in protein synthesis and bacterial physiology. Under normal growth conditions, the binding of ObgE to pre-50S subunits prevents defective subunits from being engaged in translation, thereby minimizing the chance of inefficient and faulty protein synthesis. However, under stress conditions, when ppGpp level increases in the cell, ppGpp bound ObgE not only delays the maturation of the 50S subunit, but also sequesters a large number of mature 50S subunits from taking part into translation, thereby lowering the number of active 70S ribosomes and thus, regulating the rate of total protein synthesis in the cell. Given the universal distribution of Obg proteins and (p)ppGpp system in bacteria and eukaryotic organelles [44], the action of ObgE might represent a conserved regulatory mechanism on translation in response to fluctuations in cellular energy level caused by nutrient availability.

It must be noted that in bacteria both (p)ppGpp synthetase RelA and hydrolase SpoT are associated with the ribosome, and especially, the (p)ppGpp production by RelA is well documented to be strictly dependent on deacylated A-site tRNA on the 70S ribosome [45],[46]. Our structural data show that the NTD of ObgE exactly mimics the CCA-end of the A-site tRNA. Furthermore, ObgE and SpoT were shown to co-fractionate with the pre-50S fractions [5],[11]. All these pieces of information seem to suggest that ObgE might also have additional functional interplay with RelA and SpoT. Whether or not ObgE could directly act as a regulator of the (p)ppGpp pathway remains to be investigated.

### Functional Links of ObgE with Other Cellular Pathways

A handful of genetic studies have also implicated Obg proteins in other cellular processes, such as DNA replication, chromosome segregation, and other stress response pathways (reviewed in [2],[3]). Interestingly, many of these pleiotropic phenotypes associated with Obg dysfunction appear to be species-specific, and could be attributed to the high sequence diversity within the CTDs of Obg proteins (Figure S7). For example, dysfunction of ObgE in *E. coli* causes cellular defects in DNA replication and chromosome segregation [47]–[50]. Intriguingly, these mutations with defects in DNA synthesis are primarily located to the CTD and GD of ObgE. For another example, Obg proteins in *B. subtilis* and *M. tuberculosis* were demonstrated to be involved in σ^B^-controlled general stress response [51],[52], and again, the CTD of the *B. subtilis* Obg is required for the binding to the anti-σ^B^ factor RsbW [53]. However, it must be stressed that many reported functions of Obg proteins are not independent of the ribosome or (p)ppGpp-mediated pathways. The DNA replication is known to be regulated by (p)ppGpp [54], and regulators of the σ^B^-dependent general stress response in *B. subtilis* also appear to be ribosome-associated [55],[56]. Our structural data suggest that the binding of ObgE induces a conformational change on the uL11-NTD, resulting in the displacement of the uL11-NTD from its normal position (Figure S6). This is highly consistent with roles of uL11 as key regulators in both the stringent response in *E. coli* [46] and the σ^B^-dependent general stress response in *B. subtilis* [57]. In addition, we show that the conserved function of the ObgE-NTD is to interact with the 50S subunit, in a similar way as the A-site tRNA does, and the CTD is not required for its 50S binding and anti-association activity. Therefore, the species-specific functions of Obg proteins suggest that Obg proteins might act as a specialized translation factor, partnering, through their CTDs, with distinct players in different growth control pathways to regulate ribosome assembly and protein synthesis at given energy status [3].

## Materials and Methods

### ObgE Protein Preparation

The gene for ObgE was amplified from *E. coli* DH5α genomic DNA using PCR with the following two primers: 5-GCCATATGATGAAGTTTGTTGATGAA-3 and 5-GCGGATCCTTAACGCTTGTAAATGAA-3. The 1.17 Kb PCR products were digested by NdeI and BamHI (New England Biolabs) and ligated into the pET28a vector (Novagen). A CTD deleted construct of ObgE, including coding sequence for residues 1–340 (ObgE-NG), was similarly constructed. For site-directed mutations, pET28a-*obgE* was used as PCR templates and the following primers were designed: ObgE-K27EK31E, 5-CGCCGCGAAGAGTATATTCCGGAAGGCGGC-3, and 5-GCCGCCTTCCGGAATATACTCTTCGCGGCG-3; ObgE-R76GR82G, 5-GCAAGCGGCGACTGTACCGGTAAGGGCGGTAAA-3, and 5-TTTACCGCCCTTACCGGTACAGTCGCCGCTTGC-3; ObgE-R136GR139G, 5-TCCGTTAACGGTACACCGGGGCAGAAAACC-3, and 5-GGTTTTCTGCCCCGGTGTACCGTTAACGGA-3 (mutated bases were underlined). The PCR products were digested with DpnI (New England Biolabs) to remove the template. The mutant of ObgE-K27EK31E/R76GR82G/R136GR139G was generated similarly using double-mutant plasmids as templates.

*E. coli* BL21 (DE3) cells transformed with wild type pET28a-*obgE* were grown in 1.0 liter LB medium at 37°C to OD600 of approximately 0.6 to 0.8. Protein expression was induced with 1 mM isopropyl-β-D-thiogalactopyranoside (IPTG) at 30°C for 5 h. Cells were collected at 5,000 rpm in a JLA 10.500 rotor (Beckman Coulter) for 10 min and resuspended in 40 ml lysis buffer (20 mM Tris-HCl [pH  = 7.5], 500 mM NaCl and 50 mM imidazole). Cells were lysed by sonication, and clarified lysates were obtained by centrifugation at 15,000 rpm in a JA 25.50 (Beckman Coulter) for 30 min. Lysates were loaded onto a Ni-NTA column (GE Healthcare), washed with 20 ml lysis buffer, and eluted with 10 ml elution buffer (20 mM Tris-HCl [pH  = 7.5], 500 mM NaCl and 500 mM imidazole). Protein fractions were desalted through a desalting column (HiPrep 26/10 Desalting, GE Healthcare) with desalting buffer (20 mM Tris-HCl [pH  = 7.5], 150 mM NaCl), then subjected to a RESOURCE Q column (1 ml, GE Healthcare), and eluted with a 20 ml linear gradient of NaCl from 150 to 1,000 mM. Mutant variants of ObgE (point mutations and CTD truncation) were similarly expressed and purified. Purified proteins were finally concentrated to 10 mg/ml on a 6 ml spin filter (Satorius Stedim Biotech).

### Ribosome Purification

*E. coli* 70S ribosomes were purified as described previously [58]. Purified 70S ribosomes were further centrifuged through a 10%–40% sucrose gradient with 2 mM MgCl_2_ to obtain separated 30S and 50S subunits. 50S fractions were pooled and with buffer changed to Binding buffer (20 mM Tris-HCl [pH  = 7.5], 100 mM NH_4_Cl, 10 mM MgCl_2_).

### Cell Growth and Ribosome Profile Analysis

*E. coli* cells of the BL21 strain, with or without the pET28a-*obgE* were grown at 37°C to OD600 of ∼0.5. The cell cultures were diluted to a series of concentrations (10^−3^, 10^−4^, 10^−5^, 10^−6^, and 10^−7^). 2 µl of each dilution was dropped on LB plate (1 mM IPTG) and incubated at 37°C for 10 h.

*E. coli* cells of BL21 strain with or without pET28a- *obgE* were grown at 37°C to OD600 of ∼0.5, and 1 mM IPTG was added to both cultures. After 5 h incubation at 30°C, cells were lysed by sonication, and clarified at 15,000 rpm in a JA 25.50 (Beckman Coulter) for 30 min. Equal amounts of cell extracts were loaded gently onto the top of a12 ml 10% to 40% sucrose gradient in Binding buffer, and the gradients were centrifuged in a SW41 rotor (Beckman Coulter) for 3.5 h at 39,000 rpm and 4°C. Gradients were analyzed using a Teledyne Isco fractionation system (Teledyne Isco).

### Co-sedimentation Assay

Each reaction contained a mixture of equal amounts of purified 50S subunits and His-ObgE (∼30 pmole, final concentration 1 µM) in the absence or presence of 0.5 mM GTP, GMPPNP, GDP (Sigma-Aldrich), or ppGpp (TriLink BioTechnologies). After incubation in Binding buffer for 10 min at 37°C, samples were gently loaded onto the top of 150 µl 33% sucrose cushion in Binding buffer and centrifuged at 330,000 *g* at 4°C for 4 h in a TLA120.1 rotor (Beckman Coulter). The supernatants were rapidly removed and the pellets were resuspended with 20 µl of Binding buffer. 10 µl of resolved pellets were loaded onto a 12% SDS-PAGE and the presence of His-ObgE was examined by Western blot analysis using a mouse anti-His antibody as the primary antibody, and a goat anti-mouse IgG (coupled to horseradish peroxidase) as the secondary antibody.

### 70S Ribosome Dissociation Experiment

Each reaction contained a fixed amount of 70S ribosomes (90 pmole, final concentration 1 µM), with varying amounts of ObgE or ObgE-NG, in the absence or presence of 2 mM GTP, GMPPNP, GDP, or ppGpp. The mixtures were incubated at 37°C for 10 min, loaded onto the top of a 10%–40% sucrose gradient in Binding buffer, and centrifuged in an SW41 rotor (Beckman Coulter) for 3.5 h at 39,000 rpm and 4°C. Gradients were analyzed using a Teledyne Isco fractionation system (Teledyne Isco). In Figure 1D, fractions were treated with 20% trichloroacetic acid at 4°C overnight. The pellets were isolated by centrifugation, resolved by SDS-PAGE, and then examined by Western blot analysis.

### Kinetics of Ribosomal Subunit Association

>All translation components were from *E. coli* and purified as previously described [18],[19]. Two reaction mixes were prepared in HEPES polymix buffer (pH 7.5) [18]. Mix A contained either only 30S subunit (0.5 µM) (referred to as “naked subunit”) or a 30S-preinitiation complex (30S-preIC) containing 30S (0.5 µM), 2 µM of each of XR7 mRNA encoding MLL, fMet-tRNA^fMet^ initiation factors 1 and 2 (IF1 and IF2), and GTP (100 µM). Mix B contained 50S subunit (0.5 µM) alone, with ObgE or ObgE-NG in varying concentrations (0.5–20 µM). After 5 min incubation at 37°C, equal volumes of mix A and B were mixed rapidly in a stopped flow instrument equipped with fluorescence detector set at 37°C. The extent of 70S formation was monitored by following the increase in Rayleigh light scattering at 425 nm and the observed rates (*k*_obs_) were derived by fitting the data using subunit association model described in [59]. The rates of subunit association were plotted as a function of final concentration of ObgE (or ObgE-NG). The *Ki* values were estimated from the midpoint of the curves fitted with hyperbolic equation. To check the effect of guanine nucleotides, naked subunit association was followed in the absence or presence GTP and ppGpp (100 µM) in the 50S mix containing ObgE (2.5 µM).

### Dipeptide Formation

The formation of fMet-Leu (ML) dipeptide was followed starting either from 30S-preIC or 70S-initiation complex (70S-IC). The initiation complexes were prepared by incubating 30S subunit or 70S ribosome (1 µM) with XR7 mRNA encoding MLL (1 µM), f[^3^H]Met-tRNA^fMet^ (2 µM), IF1 (1 µM), and IF2 (2 µM) at 37°C for 10 min. In parallel, an elongation mix was prepared containing leu-tRNA synthetase (0.5 µM), tRNA^Leu^ (10 µM), leucine (0.2 mM), EF-Tu (10 µM), and EF-Ts (5 µM). For dipeptide formation starting from 30S-preIC, 50S (1 µM) was added in the elongation mix. Both mixes were prepared in HEPES polymix buffer and contained GTP (1 mM), ATP (1 mM), phosphoenol pyruvate (10 µM), pyruvate kinase (50 µg/ml), and myokinase (2 µg/ml). For specific reactions ObgE (10 µM) was incubated with the elongation mix at 37°C for 10 min. Equal volumes of the initiation and the elongation mixes were rapidly mixed in a quench-flow instrument (RQF-3; KinTek Corp.). After definite time intervals the reactions were quenched, precipitated, and the peptides were analyzed by RP-HPLC as described in [18].

### *In Vitro* Translation Assay using the PURExpress System

The PURExpress *in vitro* protein synthesis kit (New England Biolabs, E6800S) was used for analyzing the effect of ObgE on translation. Transcription and translation components from the kit were mixed with purified ObgE, in a ribosome to ObgE ratio of 1∶1, 1∶2, 1∶5, 1∶10, or 1∶20. Reactions were started by adding a plasmid DNA template of dihydrofolate reductase (DHFR), and carried out at 37°C for 1 or 2 h and terminated on ice. The reaction mixtures were separated by 12% SDS-PAGE to examine the production of DHFR.

### GTPase Activity Assay

7-methyl-6-thioguanosine (MESG) assay [60] was used to determine the GTPase activity of ObgE by measuring the absorbance increase at 360 nm. In this system, the inorganic phosphate (Pi) release during GTP hydrolysis by ObgE was quantified by a coupled enzyme reaction, in which a purine nucleoside phosphorylase (PNPase) and its chromogenic substrate MESG were used. The 1.6 ml reaction system contained 100 mM MOPS (pH  = 7.0), 100 mM NaCl, 10 mM MgCl_2_, 100 µM MESG, 0.1 mg/ml PNPase, and 7.5 µM GTP. Reactions were initiated by adding ObgE to a final concentration of 3.5 µM and carried out at 25°C. The time courses of absorbance change were monitored using a Pharmacia Ultraspec III spectrometer and the Pi release was estimated with the molar extinction coefficient 11,200 M^−1^cm^−1^ for a phosphate-dependent reaction at 360 nm and pH 7.0. To determine whether the 50S subunit could simulate the GTP hydrolysis by ObgE, increasing amounts of the 50S subunits (0.02 µM, 0.1 µM, 0.69 µM, 1 µM) were mixed with 1 µM ObgE. The initial rates of reactions were calculated from the linear parts of the progress curves obtained. Nonenzymatic GTP hydrolysis was corrected by measuring the control reaction in the absence of ObgE.

### Cryo-Electron Microscopy

To reconstitute the 50S·ObgE·GMPPNP complex, purified 50S subunits (50 nM) were incubated with ObgE in a ratio of 1∶80 in the presence of 2 mM GMPPNP for 10 min at 37°C. Aliquots (5 µl) of samples were applied to carbon-coated Quantifoil 2/2 grids (Quantifoil Micro Tools GmbH), and cryo-grids were prepared as previously described [58]. The specimen was examined on an FEI Titan Krios at 300 kV at liquid-nitrogen temperature. Images were recorded on an FEI eagle CCD camera (4K × 4K) under low dose conditions (∼20 e−/Å^2^), using a nominal 59,000× magnification (effective pixel size of 1.502 Å). The data collection was performed with the software AutoEMation [61].

### Image Processing

Micrographs were processed following standard reference projection matching procedures using SPIDER [62] with some modifications. Particles were first picked using a method based on a locally normalized cross-correlation function [63] with a 256×256 window size, subjected to correspondence analysis and then manually verified [64]. Due to the sub-stoichiometric binding of ObgE, all particles (223,274 in number) were first classified in two groups, according to the presence or absence of ObgE on the 50S subunit using a modified supervised classification method (Figure S9) [65]. The resulting 188,814 ObgE-containing particles were further applied to another round of 3D classification using RELION [66]. The particles were finally split into four groups in 30 iterations using a final angle sampling of 1.8 degree. One of the four groups, which displays the highest ObgE occupancy, with a total particle number of 102,814, was used for final refinement (Figure S10). The refinement was performed using RELION [66] with the final sampling angle of 0.1 degree. The final resolution was reported by gold-standard FSC calculations [67], as 5.5 Å according to FSC 0.143 criterion (Figure S11). Amplitude correction using the B-factor sharpening approach [68] was applied to the final volume. Local resolution map was calculated using the *blocres* program of the Bsoft package [69].

### Atomic Model Building

The atomic model of the *E. coli* ObgE was built with MODELLER [70], using the *B. subtilis* and *Thermus thermophiles* Obg crystal structures (PDB IDs 1LNZ and 1UDX) [10],[17] as templates. A crystal structure of the 50S subunit (PDB ID 3OFC) [71], NTD and GD of the ObgE model were first manually docked as rigid bodies into the cryo-EM density map. The flexible fitting was performed using the molecular dynamics flexible fitting approach [72]. PyMol [73] and Chimera [74] were used for graphic visualization and figure preparation.

### Accession Codes

Cryo-EM map of the 50S·ObgE complex has been deposited in the EMDataBank (EMD-2605). The atomic model has been deposited in the Protein Data Bank (4CSU).

## Supporting Information

Figure S1 **ObgE-NG promotes the dissociation of 70S ribosomes.** (A) ObgE-NG binds to the 50S subunit with comparable affinity as full-length ObgE does. The co-sedimentation experiments were performed with combinations of different components. Experimental groups contained ∼1 µM 50S subunits and 50-fold ObgE or ObgE-NG in the presence of 2 mM GMPPNP. Both the pellets and supernatants were resolved by SDS-PAGE. (B) Dissociation of 70S ribosomes (1 µM) by varying amount of ObgE-NG (from 5- to 50-fold excess), in the presence of GDP (2 mM). (C) Dissociation of 70S ribosomes (1 µM) by 30-fold excess of ObgE-NG, in the presence of GDP, GTP, GMPPNP, or ppGpp (2 mM).
(TIF)

Figure S2 **ObgE inhibits translation** ***in vitro*** **.** Coupled *in vitro* transcription and translation system was programmed without (lane 1) or with (lane 2–7) the DHFR DNA template. Lane 3–7, increasing amounts of purified ObgE (in 1, 2, 5, 10, or 20-fold excess) were added to the system to test the effect of ObgE on protein translation. Samples were taken at 1-hour and 2-hour time points, and resolved by SDS-PAGE. The bands of ObgE and DHFR are indicated.
(TIF)

Figure S3 **The effect of overexpression of ObgE on the cell growth and ribosome profile.** (A) Spot assay of the *E. coli* BL21 and BL21-*obgE* overexpression strains. 1 mM IPTG was added in the culture plates. (B). Time-course growth curve of the *E. coli* BL21 (▪) and BL21-ObgE overexpression (•) strains. 1 mM IPTG was added at 4-hour time point. (C and D) *In vivo* ribosome profiles of the *E. coli* BL21 (C) and BL21-*ObgE* (D) strains after IPTG induction. The fractions of the 30S, 50S, 70S, and polysomes are labeled.
(TIF)

Figure S4 **Comparison of the binding position of ObgE with translational GTPases on the 50S subunit.** Superimposition of ObgE (purple) with the atomic structures of the 50S subunit bound with (A) IF2 (cyan) (PDB 1ZO3) [25], (B) EF-G (yellow) (PDB 2WRI and 2WRJ) [26], (C) EF-Tu (orange) (PDB 2WRN and 2WRO) [27], and (D) RF3 (wheat) (PDB 3SFS and 3SGF) [29].
(TIF)

Figure S5 **The atomic model of the** ***E. coli*** **ObgE.** (A) Segmented cryo-EM density map of ObgE, superimposed with fitted atomic model. (B) The atomic model of the *E. coli* ObgE is shown in cartoon representation. The GD, NTD β-stranded base, and the NTD left-handed helix protrusion are colored purple, orange, and yellow, respectively. Loop 1, loop 2, and loop 3 (numbered from the N-terminus) are colored red, dark blue, and green, respectively. (C) The crystal structure of *T. thermophiles* Obg (PDB ID 1UDX, cyan) [17], the homology model of ObgE (wheat), and the 50S-bound model of ObgE (purple) are superimposed, using the GD as the reference for alignment.
(TIF)

Figure S6 **Interaction of the ObgE-GD with the GTPase associated center.** Intersubunit view of the 50S·ObgE·GMPPNP complex, showing the interactions between the ObgE-GD and the GAC of the 50S subunit. H43 and H44 of the GAC are colored red, with A1067 shown in cartoon representation. The NTD of uL11 is colored orange with proline 21 (P21) and proline 22 (P22) shown in stick model (cyan). Switch II of ObgE-GD is colored grey.
(TIF)

Figure S7 **Sequence alignment of Obg proteins from different species.** Sequences of Obg proteins from *B. subtilis* (NP_390670.1), *T. thermophilus* (YP_145047.1), *E. coli* (WP_021552409.1), *S. cerevisiae* (NP_012038.2), *A. thaliana* (NP_197358.2), and *H. sapiens* (NP_056481.1), were aligned using MUSCLE [76]. Residues of loop 1, loop 2, loop 3, switch I, switch II, and CTD are indicated by colored boxes. The five, G1–G5, motifs of the GTPase domain are also labeled. Conserved lysine and arginine residues of ObgE that show specific interactions with the 23S rRNA are labeled with asteroids.
(TIF)

Figure S8 **Relative position of ObgE and modification sites of RrmJ, RluD, and RluC.** Modification sites of RrmJ, RluD, and RluC are colored red, pink, and yellow, respectively, and displayed in cartoon representation. The 23S rRNA, A-loop, P-loop, and ObgE are colored blue, cyan, lime, and purple, respectively.
(TIF)

Figure S9 **Supervised classification of particles based on the absence of presence of ObgE on the 50S subunit.** All 223,274 particles were first used to reconstruct a 3D volume using standard reference projection matching technique. A 3D mask was then created by subtracting an empty 50S density map from the reconstructed map. 83 2D masks were further generated by projecting the 3D mask at an angular step of 15°. Particles were grouped into two classes, on the basis of their average densities within their respective 2D masks. A total of 188,814 particles were finally used for further refinement.
(TIF)

Figure S10 **3D classification of ObgE-bound particles.** The particles were classified into four groups, one of which with the highest ObgE occupancy was used for final refinement.
(TIF)

Figure S11 **FSC curve of the cryo-EM map.** Fourier Shell Correlation (FSC) curve of the density map of the 50S·ObgE·GMPPNP complex. The final resolution is 5.5 Å based on gold standard FSC according to 0.143 criterion.
(TIF)

## Abbreviations

cryo-EM: cryo-electron microscopy
DHFR: dihydrofolate reductase
GD: GTPase domain
NTD: N-terminal domain
CTD: C-terminal domain
ObgE: Obg protein in *Escherichia coli*
(p)ppGpp: guanosine tetraphosphate and guanosine pentaphosphate collectively
ppGpp: guanosine tetraphosphate
pppGpp: guanosine pentaphosphate
PTC: peptidyl-transfer center
SRL: sarcin-ricin loop
