# Supplementary figures and images for: Structural and Functional Insights into the Mode of Action of a Universally Conserved Obg GTPase
Source: PLoS Biol. 2014 May 20;12(5):e1001866. doi: 10.1371/journal.pbio.1001866 (PMC4028186; doi:10.1371/journal.pbio.1001866)

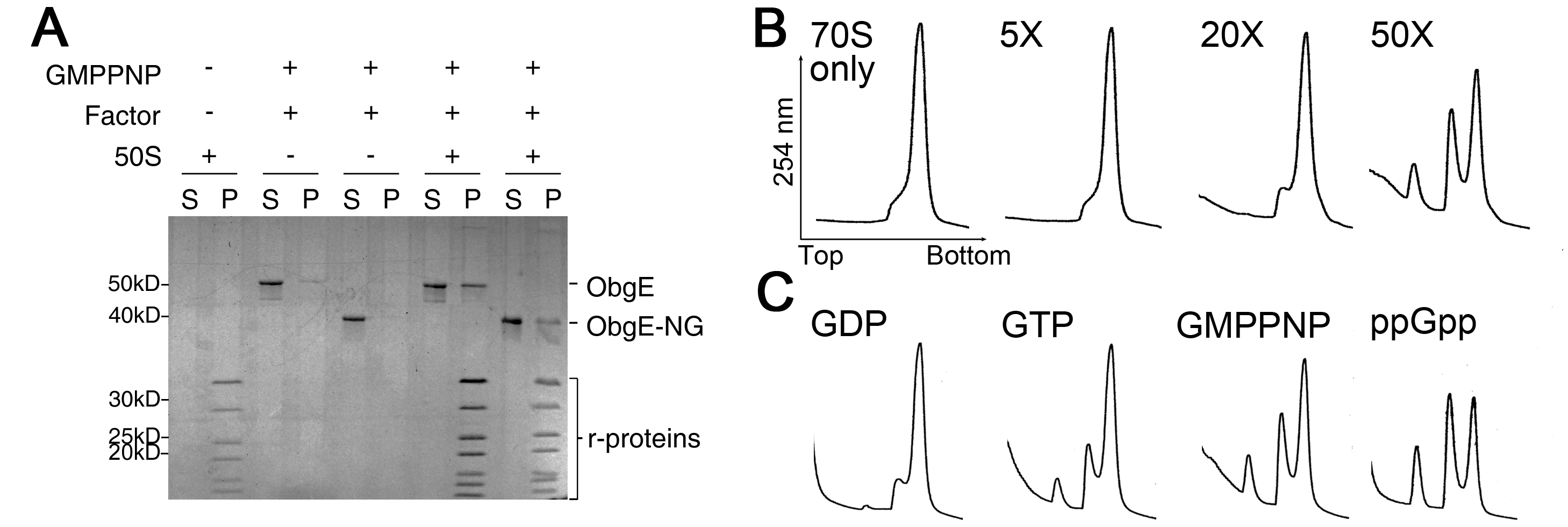

Supplement: Figure S1 — ObgE-NG promotes the dissociation of 70S ribosomes. (A) ObgE-NG binds to the 50S subunit with comparable affinity as full-length ObgE does. The co-sedimentation experiments were performed with combinations of different components. Experimental groups contained ∼1 µM 50S subunits and 50-fold ObgE or ObgE-NG in the presence of 2 mM GMPPNP. Both the pellets and supernatants were resolved by SDS-PAGE. (B) Dissociation of 70S ribosomes (1 µM) by varying amount of ObgE-NG (from 5- to 50-fold excess), in the presence of GDP (2 mM). (C) Dissociation of 70S ribosomes (1 µM) by 30-fold excess of ObgE-NG, in the presence of GDP, GTP, GMPPNP, or ppGpp (2 mM). (TIF) [file pbio.1001866.s001.tif]

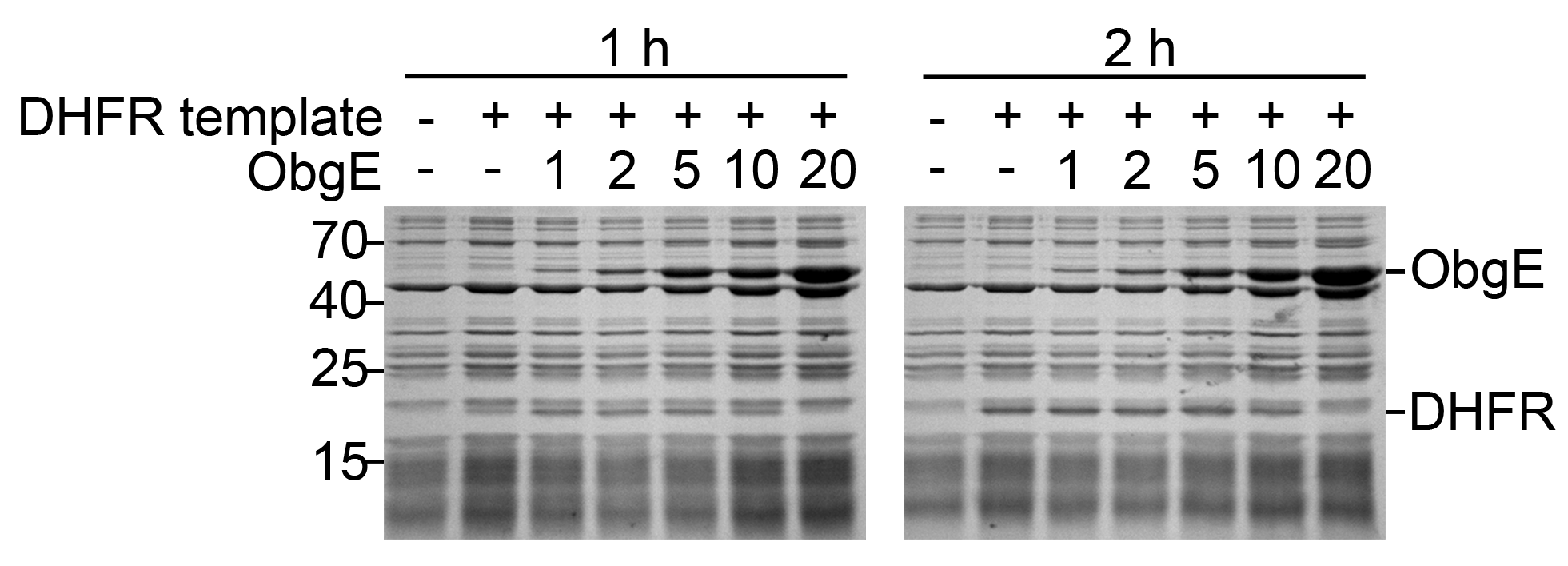

Supplement: Figure S2 — ObgE inhibits translation in vitro . Coupled in vitro transcription and translation system was programmed without (lane 1) or with (lane 2–7) the DHFR DNA template. Lane 3–7, increasing amounts of purified ObgE (in 1, 2, 5, 10, or 20-fold excess) were added to the system to test the effect of ObgE on protein translation. Samples were taken at 1-hour and 2-hour time points, and resolved by SDS-PAGE. The bands of ObgE and DHFR are indicated. (TIF) [file pbio.1001866.s002.tif]

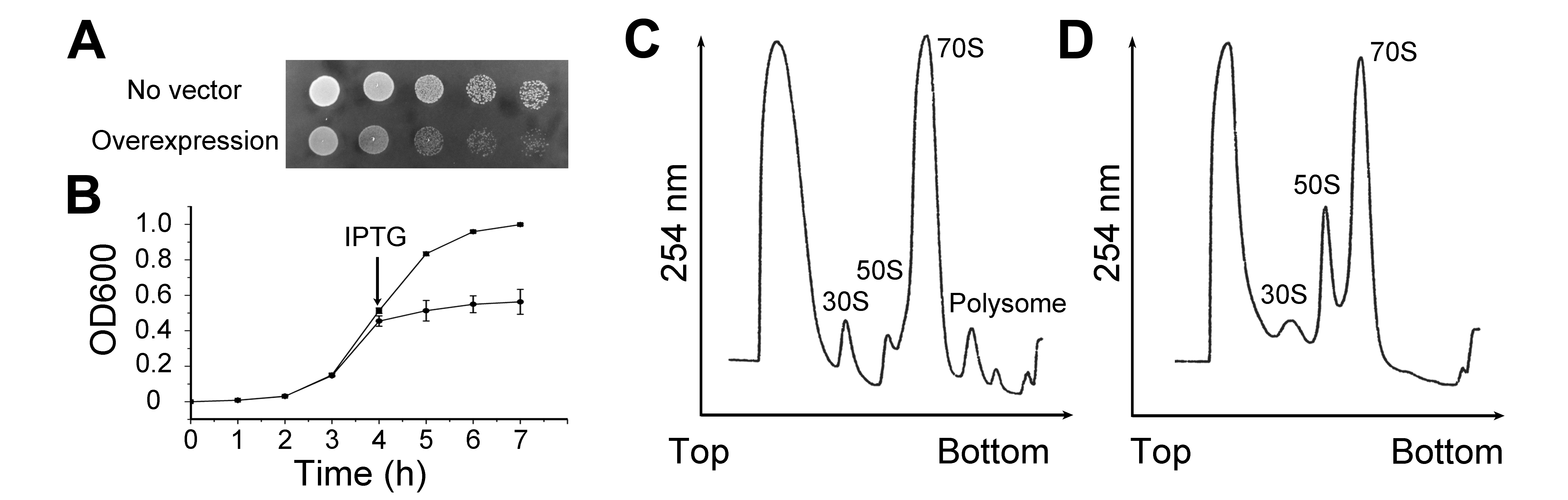

Supplement: Figure S3 — The effect of overexpression of ObgE on the cell growth and ribosome profile. (A) Spot assay of the E. coli BL21 and BL21-obgE overexpression strains. 1 mM IPTG was added in the culture plates. (B). Time-course growth curve of the E. coli BL21 (▪) and BL21-ObgE overexpression (•) strains. 1 mM IPTG was added at 4-hour time point. (C and D) In vivo ribosome profiles of the E. coli BL21 (C) and BL21-ObgE (D) strains after IPTG induction. The fractions of the 30S, 50S, 70S, and polysomes are labeled. (TIF) [file pbio.1001866.s003.tif]

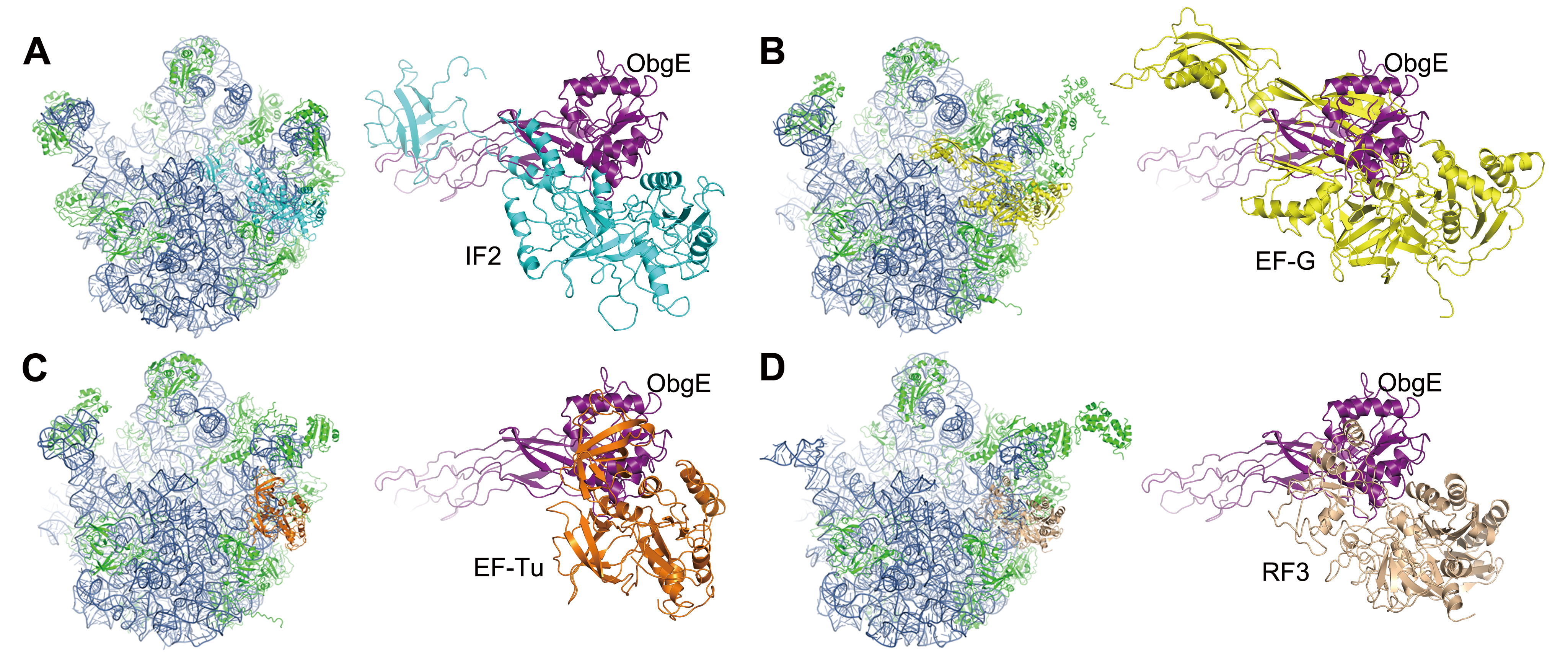

Supplement: Figure S4 — Comparison of the binding position of ObgE with translational GTPases on the 50S subunit. Superimposition of ObgE (purple) with the atomic structures of the 50S subunit bound with (A) IF2 (cyan) (PDB 1ZO3) [25], (B) EF-G (yellow) (PDB 2WRI and 2WRJ) [26], (C) EF-Tu (orange) (PDB 2WRN and 2WRO) [27], and (D) RF3 (wheat) (PDB 3SFS and 3SGF) [29]. (TIF) [file pbio.1001866.s004.tif]

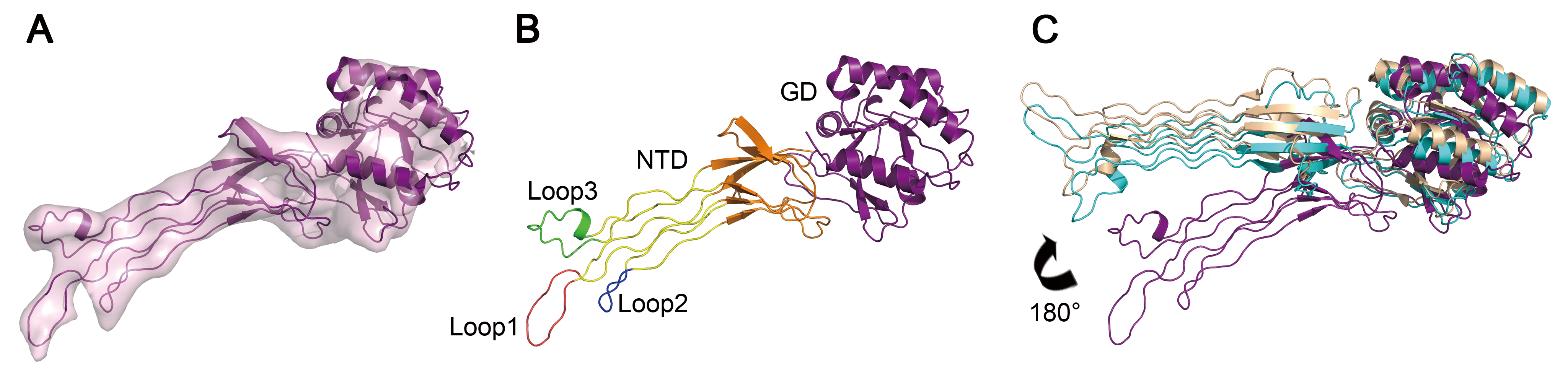

Supplement: Figure S5 — The atomic model of the E. coli ObgE. (A) Segmented cryo-EM density map of ObgE, superimposed with fitted atomic model. (B) The atomic model of the E. coli ObgE is shown in cartoon representation. The GD, NTD β-stranded base, and the NTD left-handed helix protrusion are colored purple, orange, and yellow, respectively. Loop 1, loop 2, and loop 3 (numbered from the N-terminus) are colored red, dark blue, and green, respectively. (C) The crystal structure of T. thermophiles Obg (PDB ID 1UDX, cyan) [17], the homology model of ObgE (wheat), and the 50S-bound model of ObgE (purple) are superimposed, using the GD as the reference for alignment. (TIF) [file pbio.1001866.s005.tif]

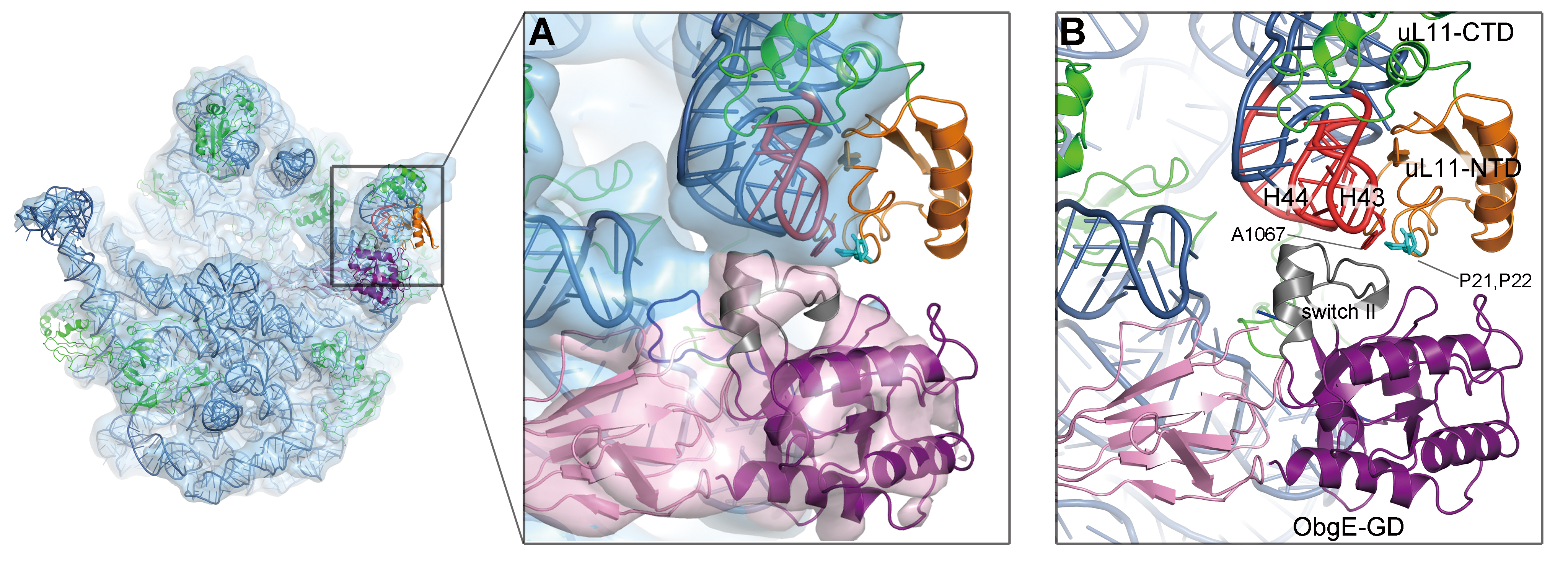

Supplement: Figure S6 — Interaction of the ObgE-GD with the GTPase associated center. Intersubunit view of the 50S·ObgE·GMPPNP complex, showing the interactions between the ObgE-GD and the GAC of the 50S subunit. H43 and H44 of the GAC are colored red, with A1067 shown in cartoon representation. The NTD of uL11 is colored orange with proline 21 (P21) and proline 22 (P22) shown in stick model (cyan). Switch II of ObgE-GD is colored grey. (TIF) [file pbio.1001866.s006.tif]

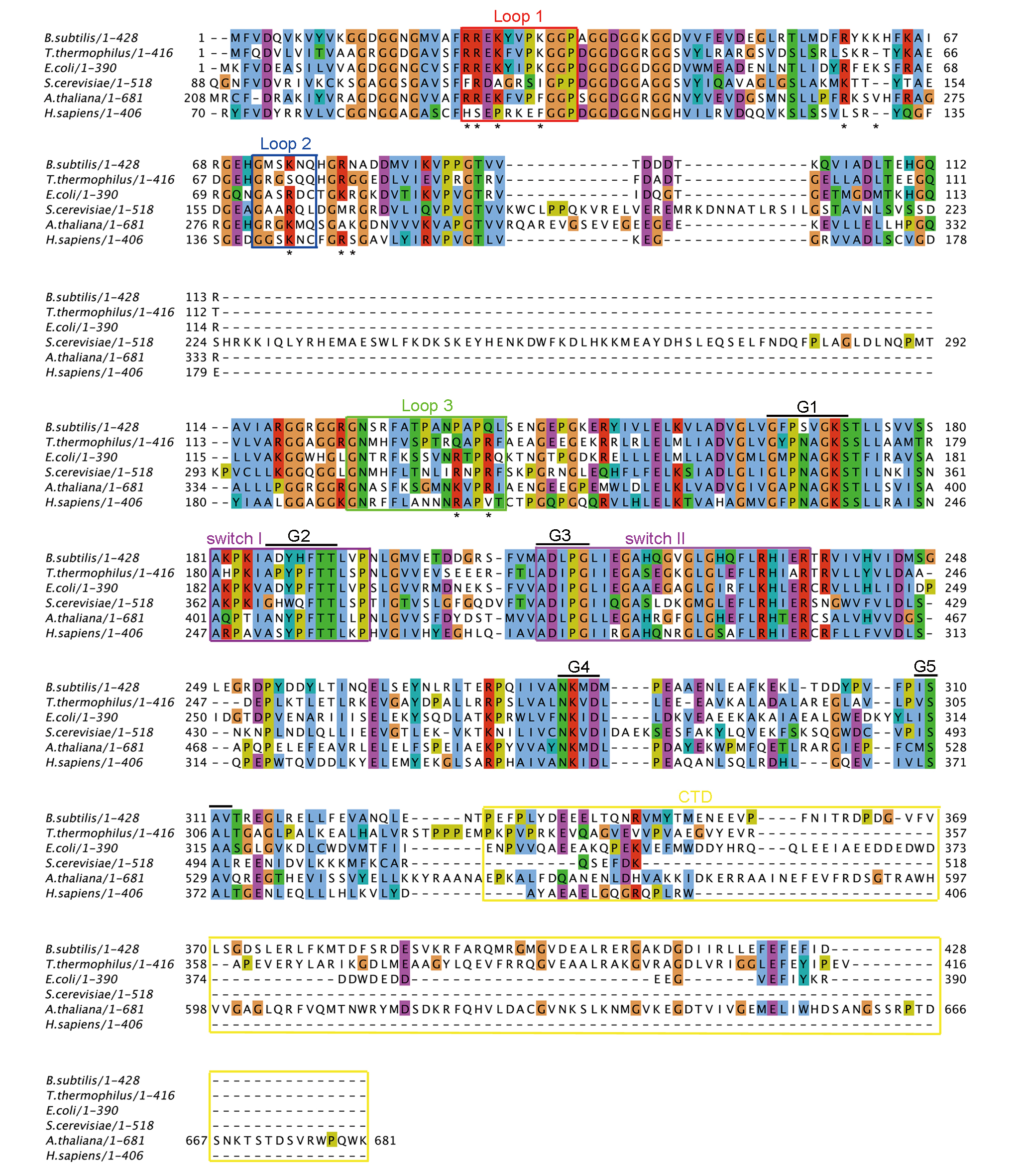

Supplement: Figure S7 — Sequence alignment of Obg proteins from different species. Sequences of Obg proteins from B. subtilis (NP_390670.1), T. thermophilus (YP_145047.1), E. coli (WP_021552409.1), S. cerevisiae (NP_012038.2), A. thaliana (NP_197358.2), and H. sapiens (NP_056481.1), were aligned using MUSCLE [76]. Residues of loop 1, loop 2, loop 3, switch I, switch II, and CTD are indicated by colored boxes. The five, G1–G5, motifs of the GTPase domain are also labeled. Conserved lysine and arginine residues of ObgE that show specific interactions with the 23S rRNA are labeled with asteroids. (TIF) [file pbio.1001866.s007.tif]

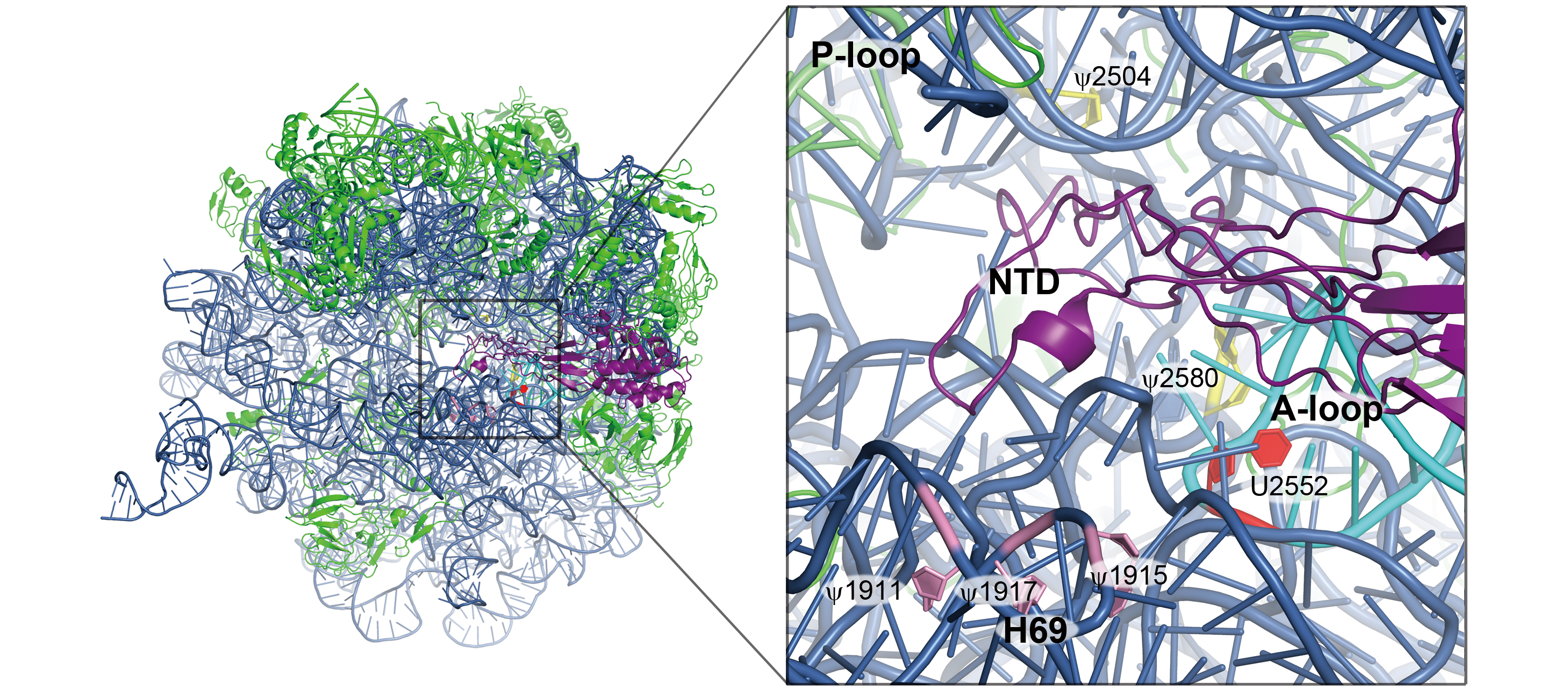

Supplement: Figure S8 — Relative position of ObgE and modification sites of RrmJ, RluD, and RluC. Modification sites of RrmJ, RluD, and RluC are colored red, pink, and yellow, respectively, and displayed in cartoon representation. The 23S rRNA, A-loop, P-loop, and ObgE are colored blue, cyan, lime, and purple, respectively. (TIF) [file pbio.1001866.s008.tif]

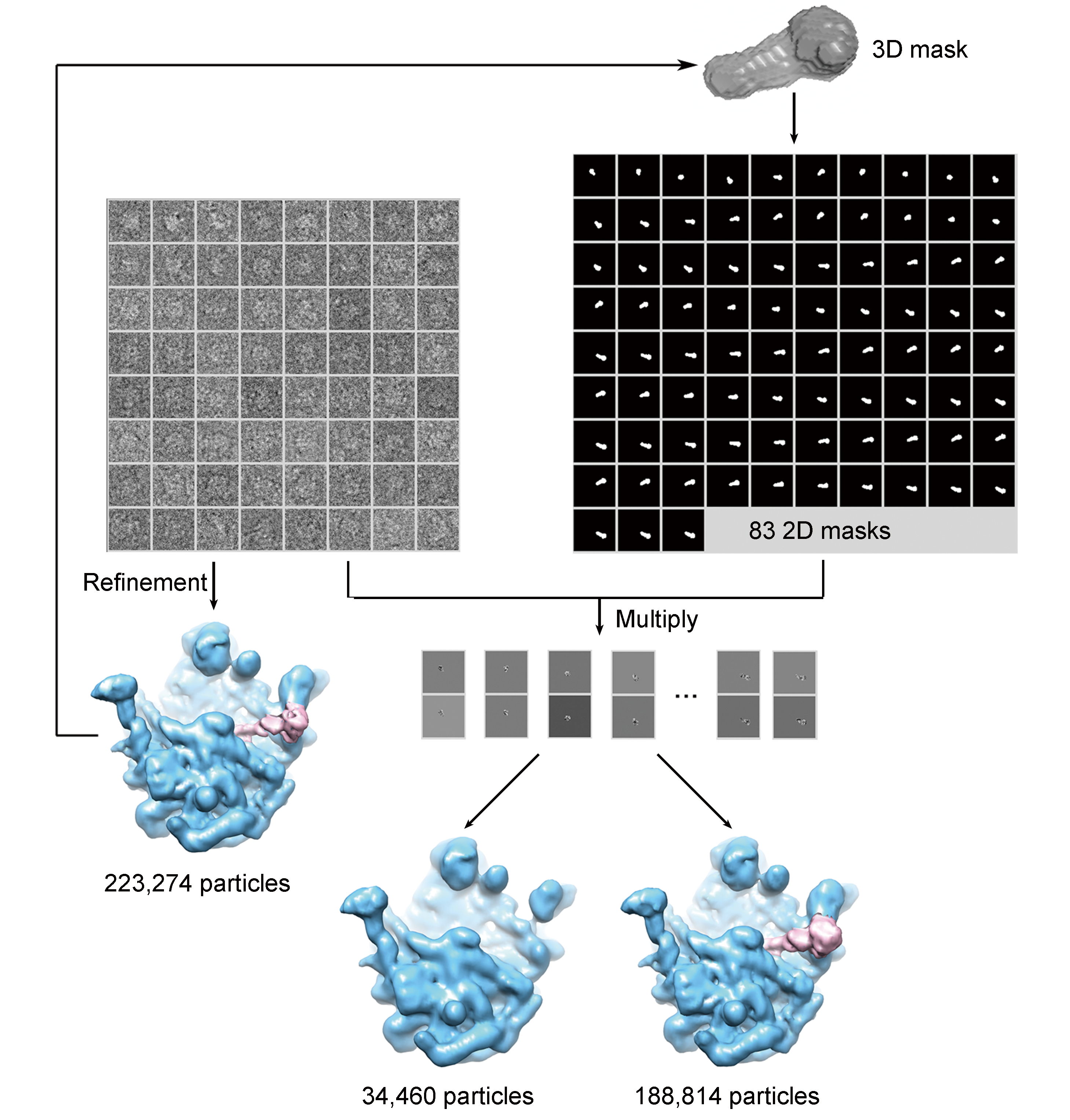

Supplement: Figure S9 — Supervised classification of particles based on the absence of presence of ObgE on the 50S subunit. All 223,274 particles were first used to reconstruct a 3D volume using standard reference projection matching technique. A 3D mask was then created by subtracting an empty 50S density map from the reconstructed map. 83 2D masks were further generated by projecting the 3D mask at an angular step of 15°. Particles were grouped into two classes, on the basis of their average densities within their respective 2D masks. A total of 188,814 particles were finally used for further refinement. (TIF) [file pbio.1001866.s009.tif]

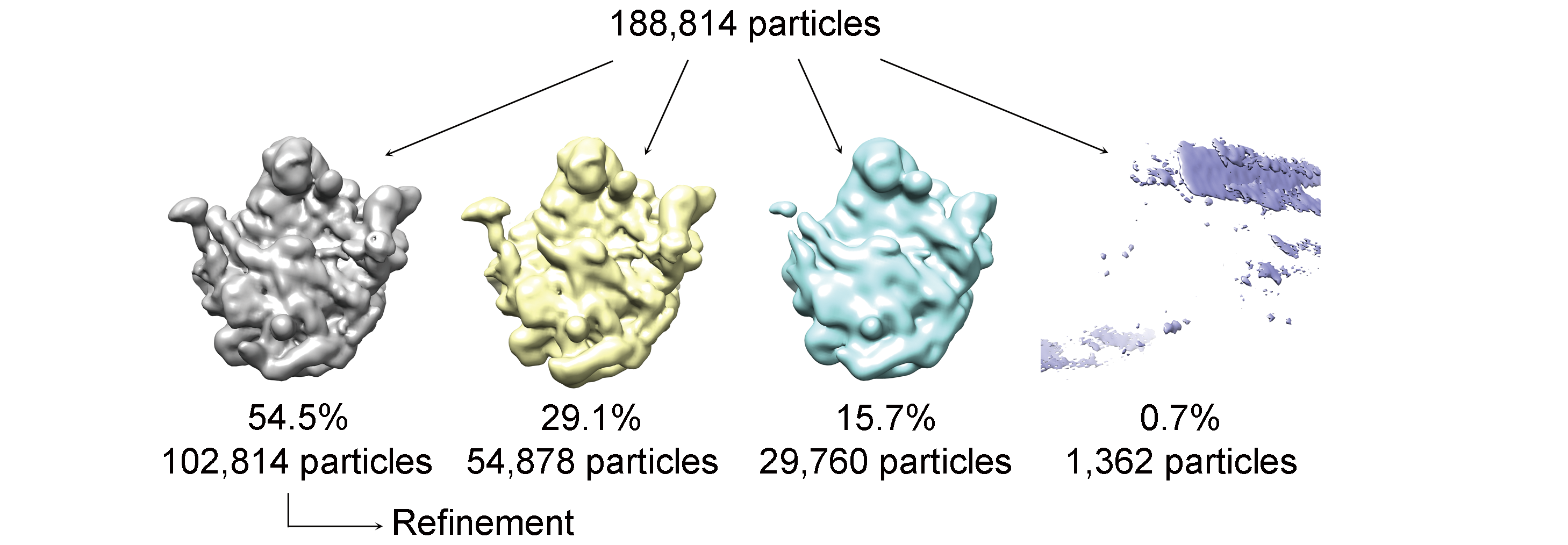

Supplement: Figure S10 — 3D classification of ObgE-bound particles. The particles were classified into four groups, one of which with the highest ObgE occupancy was used for final refinement. (TIF) [file pbio.1001866.s010.tif]

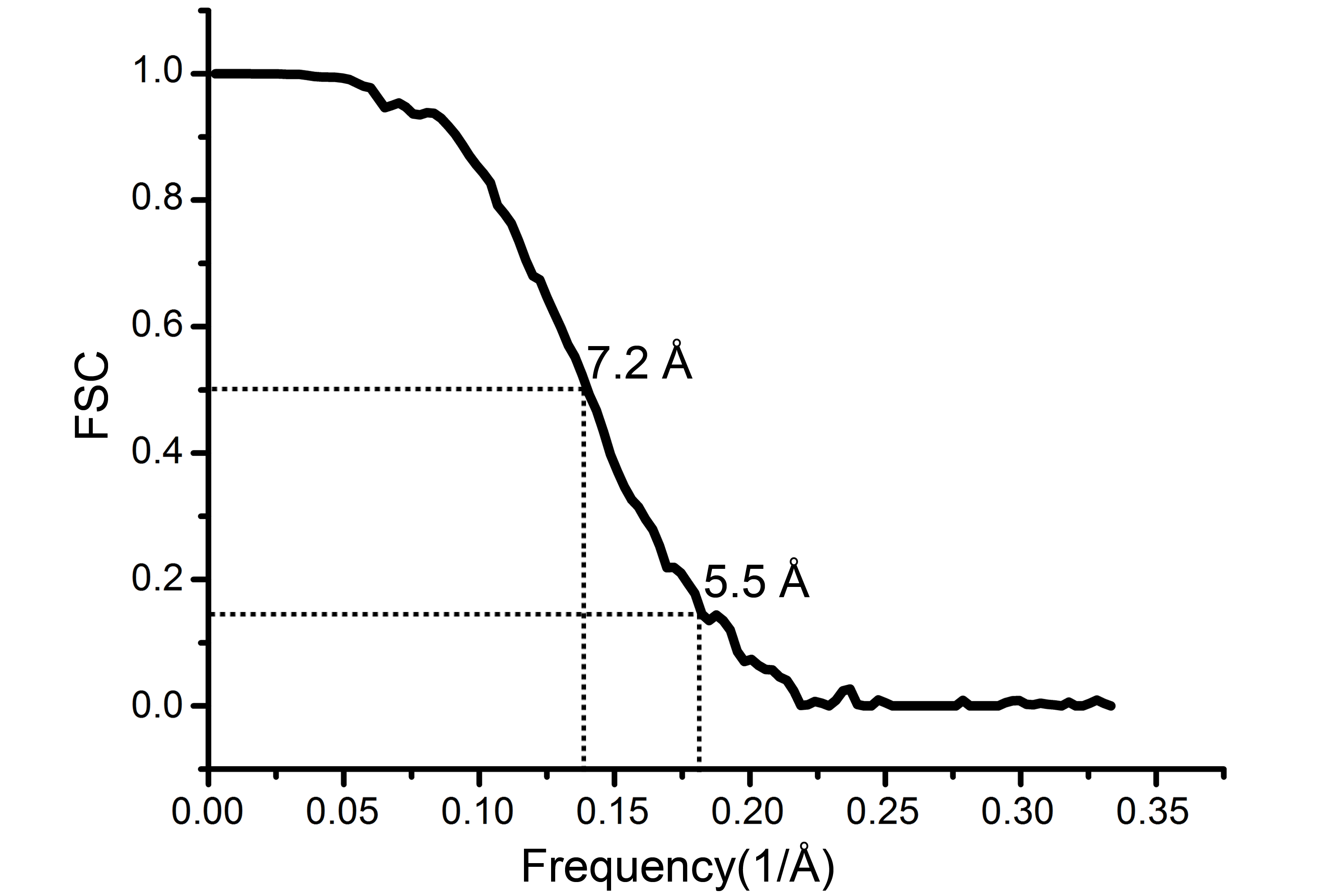

Supplement: Figure S11 — FSC curve of the cryo-EM map. Fourier Shell Correlation (FSC) curve of the density map of the 50S·ObgE·GMPPNP complex. The final resolution is 5.5 Å based on gold standard FSC according to 0.143 criterion. (TIF) [file pbio.1001866.s011.tif]
